# Supplementary figures and images for: In vivo nanoscale analysis of the dynamic synergistic interaction of Bacillus thuringiensis Cry11Aa and Cyt1Aa toxins in Aedes aegypti
Source: PLoS Pathog. 2021 Jan 19;17(1):e1009199. doi: 10.1371/journal.ppat.1009199 (PMC7846010; doi:10.1371/journal.ppat.1009199)

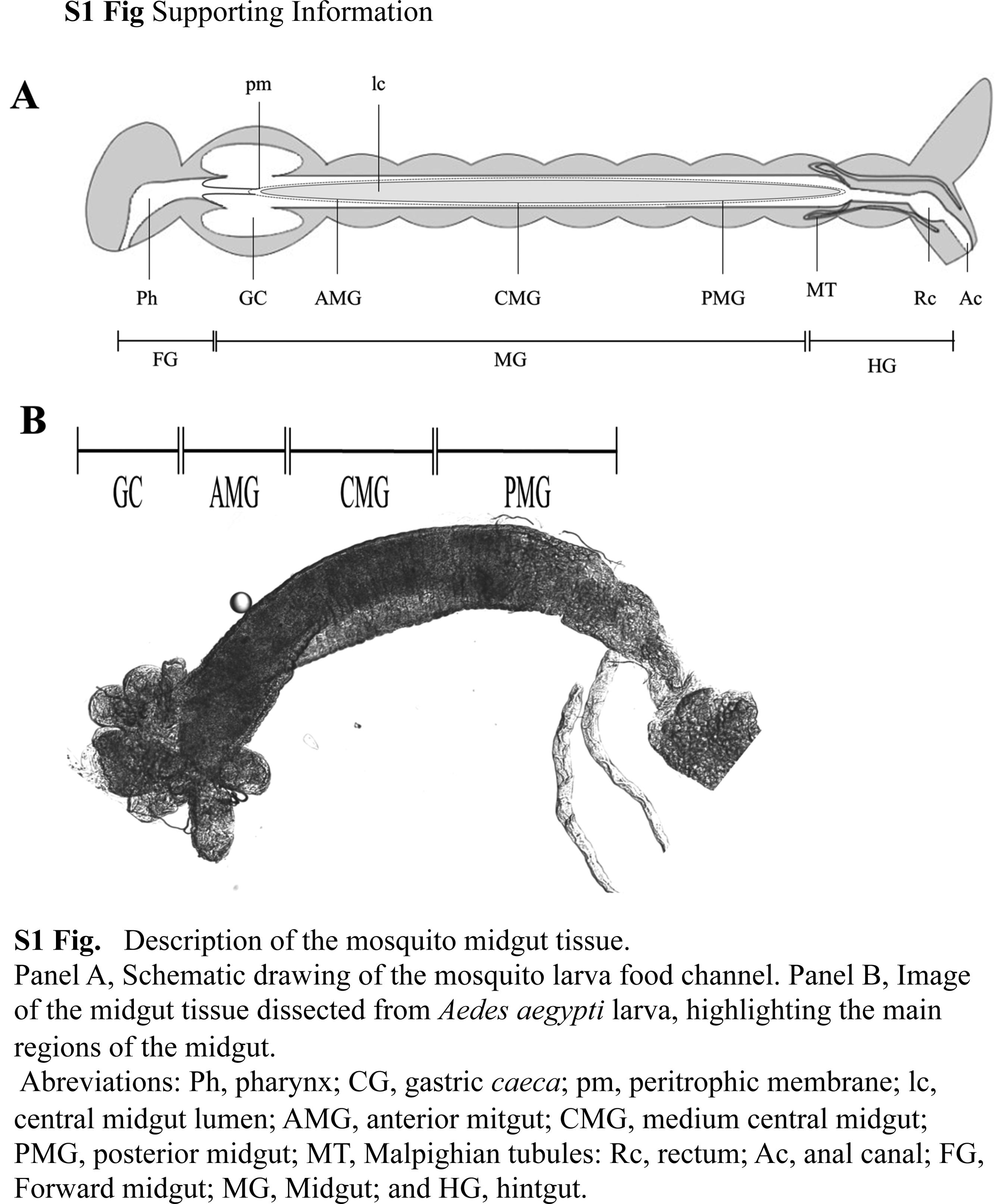

Supplement: S1 Fig — Panel A, Schematic drawing of the mosquito larva food channel. Panel B, Image of the midgut tissue dissected from Aedes aegypti larva, highlighting the main regions of the midgut. Abbreviations: Ph, pharynx; CG, gastric caeca; pm, peritrophic membrane; lc, central midgut lumen; AMG, anterior midgut; CMG, medium central midgut; PMG, posterior midgut; MT, Malpighian tubules: Rc, rectum; Ac, anal canal; FG, Forward midgut; MG, Midgut; and HG, hindgut. (TIF) [file ppat.1009199.s001.tif]

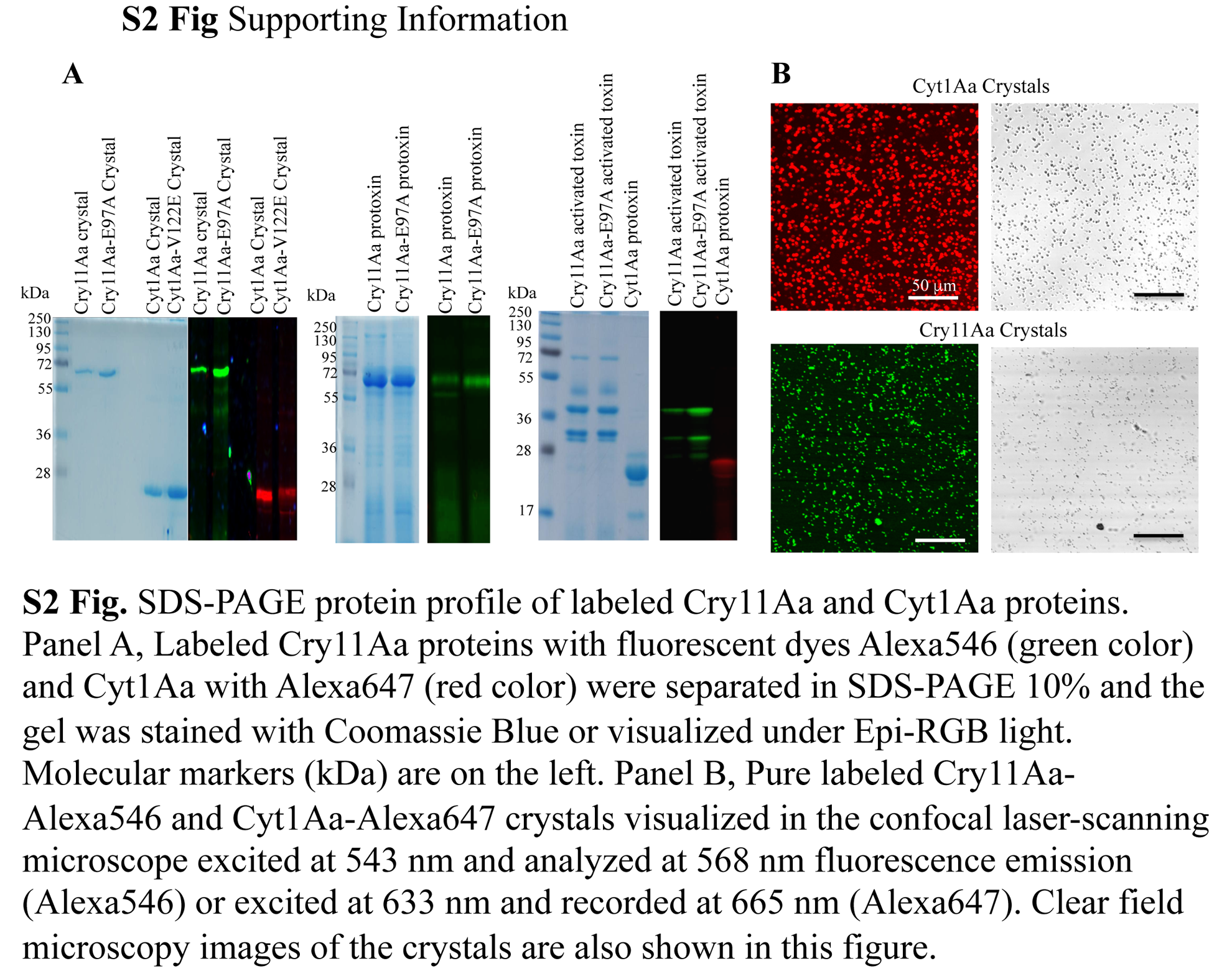

Supplement: S2 Fig — Panel A, Labeled Cry11Aa proteins with fluorescent dyes Alexa546 (green color) and Cyt1Aa with Alexa647 (red color) were separated in SDS-PAGE 10% and the gel was stained with Coomassie Blue or visualized under Epi-RGB light. Molecular markers (kDa) are on the left. Panel B, Pure labeled Cry11Aa-Alexa546 and Cyt1Aa-Alexa647 crystals visualized in the confocal laser-scanning microscope excited at 543 nm and analyzed at 568 nm fluorescence emission (Alexa546) or excited at 633 nm and recorded at 665 nm (Alexa647). Clear field microscopy images of the crystals are also shown in this figure. (TIF) [file ppat.1009199.s002.tif]

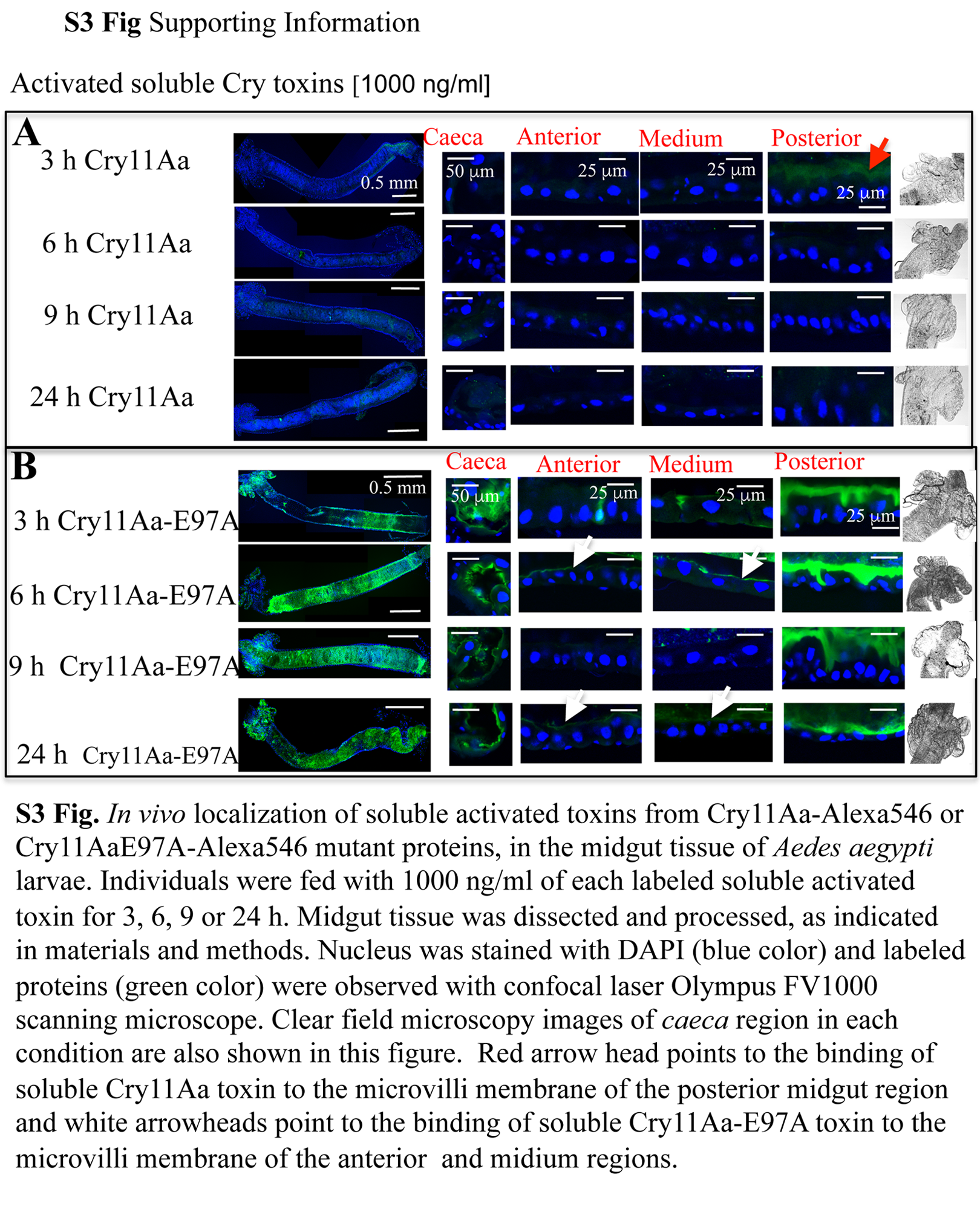

Supplement: S3 Fig — Individuals were fed with 1000 ng/ml of each labeled soluble activated toxin for 3, 6, 9 or 24 h. Midgut tissue was dissected and processed, as indicated in Materials and methods. Nucleus was stained with DAPI (blue color) and labeled proteins (green color) were observed with confocal laser Olympus FV1000 scanning microscope. Clear field microscopy images of caeca region in each condition are also shown in this figure. Red arrow head points to the binding of soluble Cry11Aa toxin to the microvilli membrane of the posterior midgut region and white arrowheads point to the binding of soluble Cry11Aa-E97A toxin to the microvilli membrane of the anterior and medium regions. (TIF) [file ppat.1009199.s003.tif]

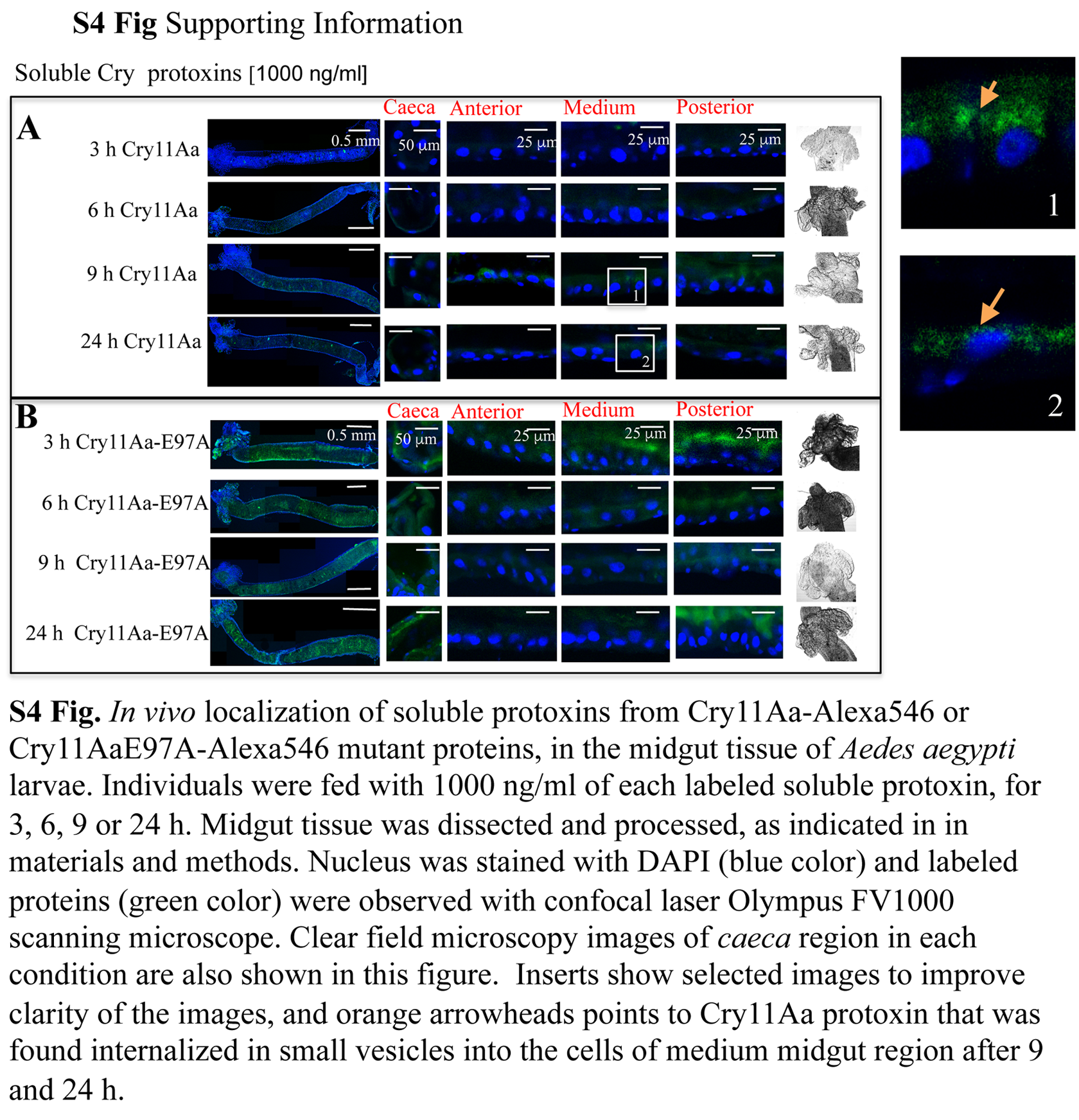

Supplement: S4 Fig — Individuals were fed with 1000 ng/ml of each labeled soluble protoxin, for 3, 6, 9 or 24 h. Midgut tissue was dissected and processed, as indicated in in Materials and methods. Nucleus was stained with DAPI (blue color) and labeled proteins (green color) were observed with confocal laser Olympus FV1000 scanning microscope. Clear field microscopy images of caeca region in each condition are also shown in this figure. Inserts show selected images to improve clarity of the images, and orange arrowheads points to Cry11Aa protoxin that was found internalized in small vesicles into the cells of medium midgut region after 9 and 24 h. (TIF) [file ppat.1009199.s004.tif]

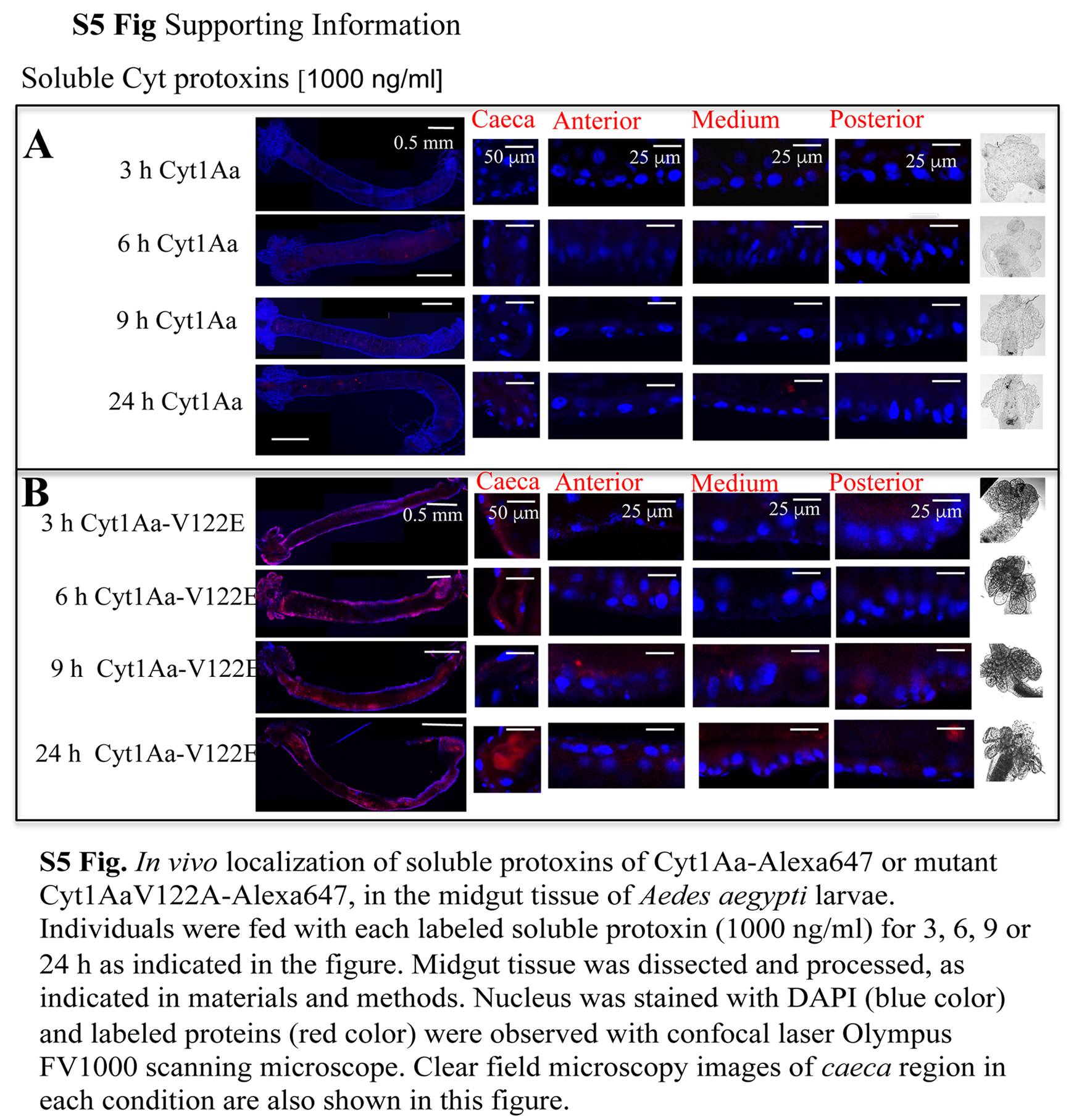

Supplement: S5 Fig — Individuals were fed with each labeled soluble protoxin (1000 ng/ml) for 3, 6, 9 or 24 h as indicated in the figure. Midgut tissue was dissected and processed, as indicated in Materials and methods. Nucleus was stained with DAPI (blue color) and labeled proteins (red color) were observed with confocal laser Olympus FV1000 scanning microscope. Clear field microscopy images of caeca region in each condition are also shown in this figure. (TIF) [file ppat.1009199.s005.tif]

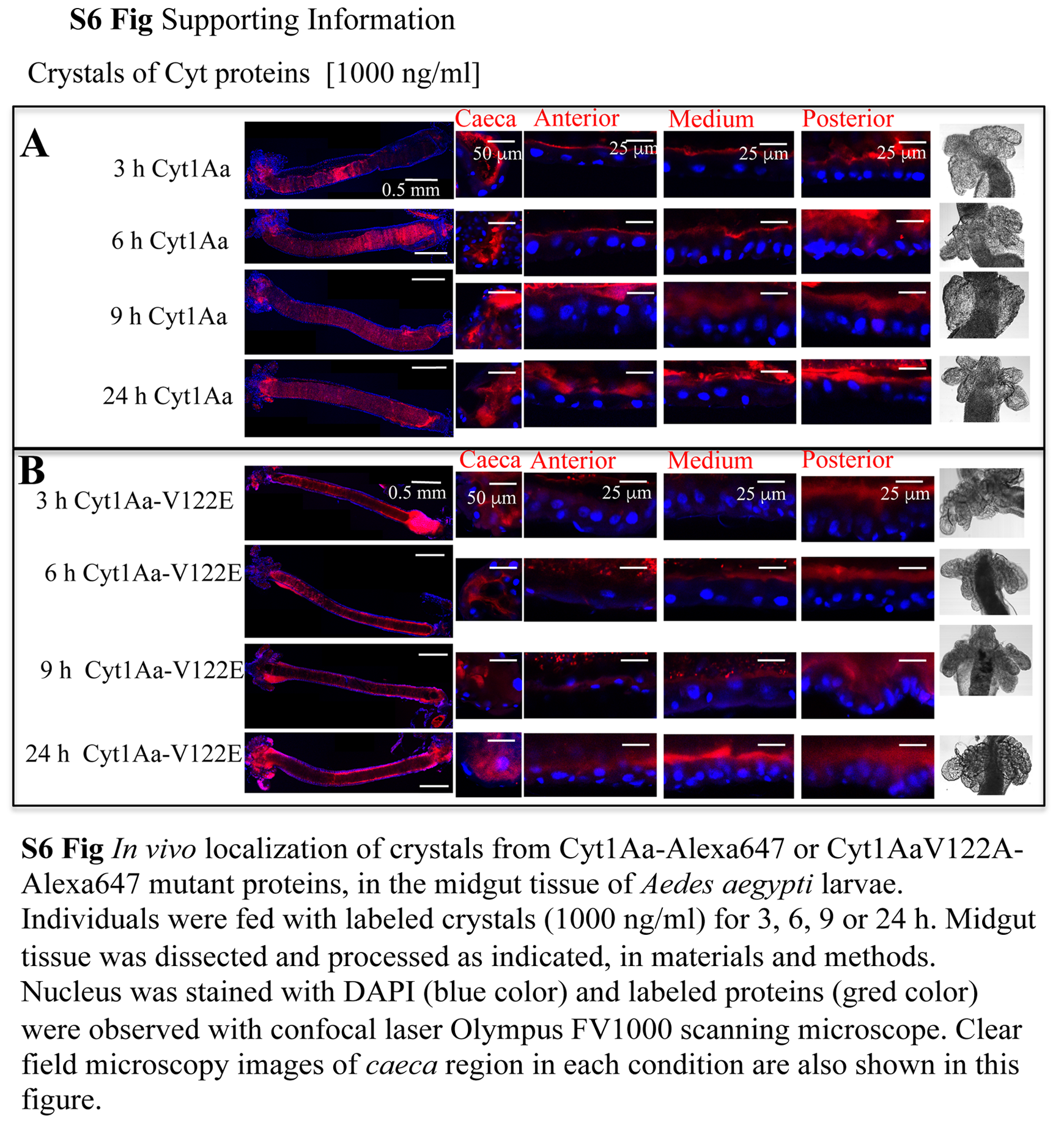

Supplement: S6 Fig — Individuals were fed with labeled crystals (1000 ng/ml) for 3, 6, 9 or 24 h. Midgut tissue was dissected and processed as indicated, in Materials and methods. Nucleus was stained with DAPI (blue color) and labeled proteins (red color) were observed with confocal laser Olympus FV1000 scanning microscope. Clear field microscopy images of caeca region in each condition are also shown in this figure. (TIF) [file ppat.1009199.s006.tif]

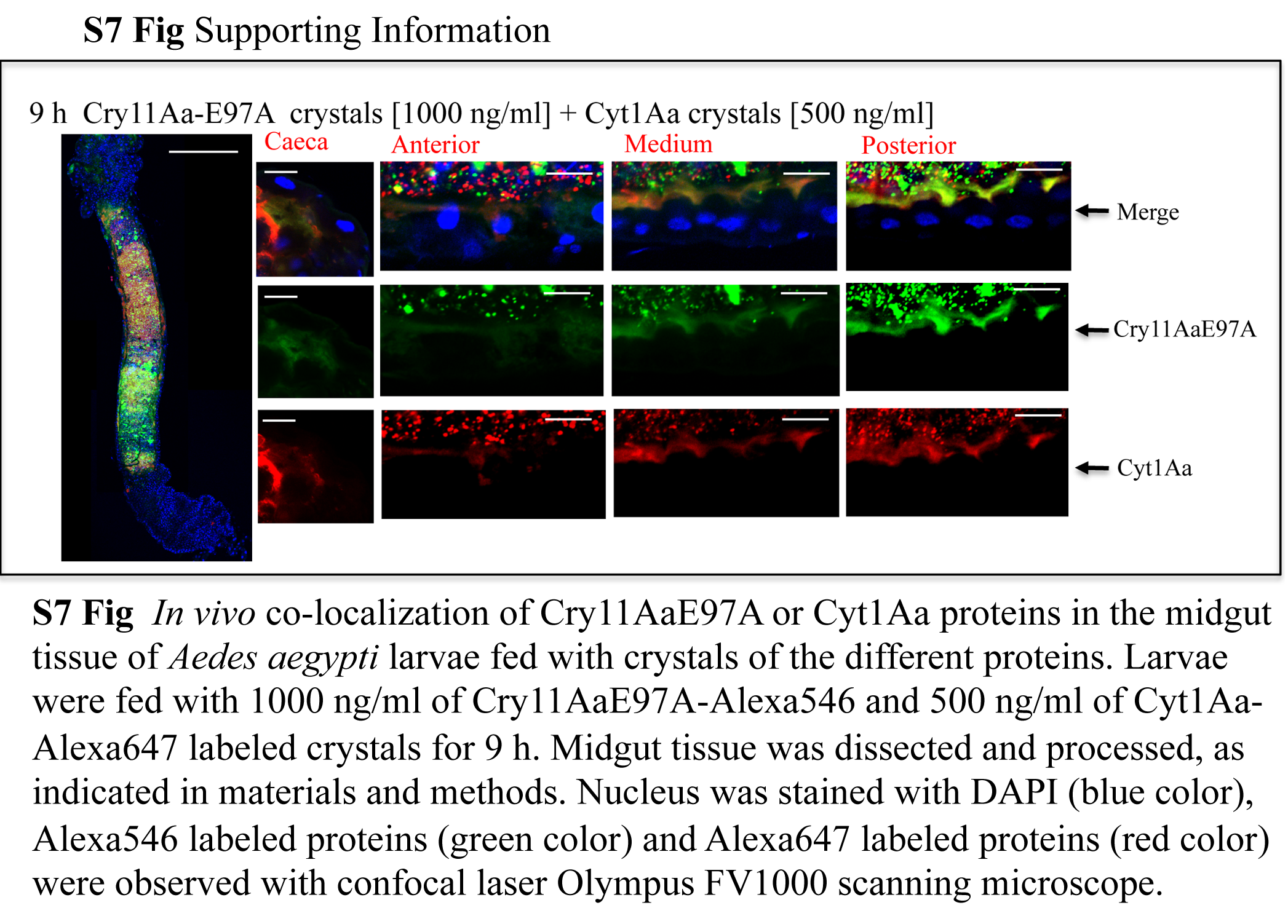

Supplement: S7 Fig — Larvae were fed with 1000 ng/ml of Cry11AaE97A-Alexa546 and 500 ng/ml of Cyt1Aa-Alexa647 labeled crystals for 9 h. Midgut tissue was dissected and processed, as indicated in Materials and methods. Nucleus was stained with DAPI (blue color), Alexa546 labeled proteins (green color) and Alexa647 labeled proteins (red color) were observed with confocal laser Olympus FV1000 scanning microscope. (TIF) [file ppat.1009199.s007.tif]

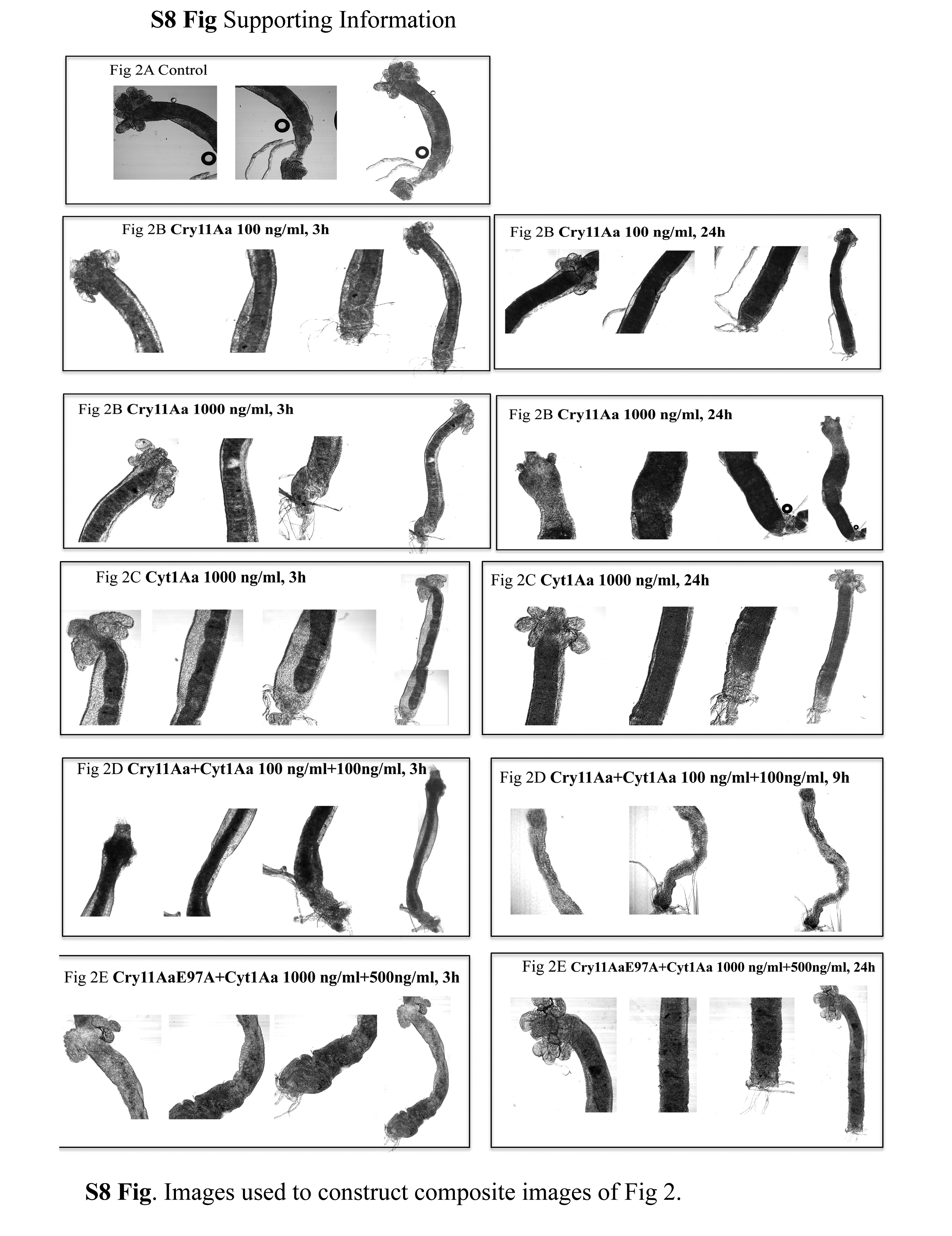

Supplement: S8 Fig — (TIF) [file ppat.1009199.s008.tif]

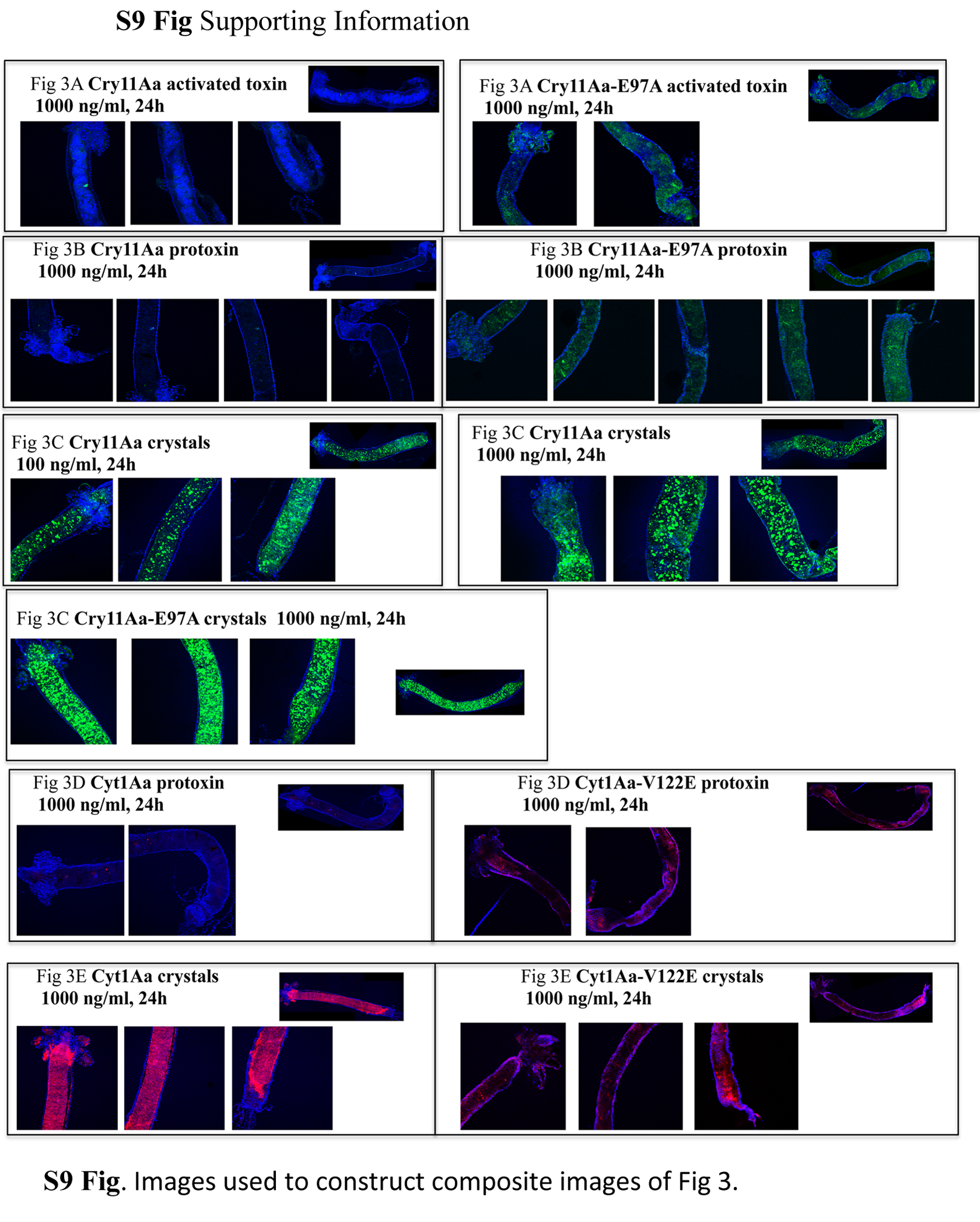

Supplement: S9 Fig — (TIF) [file ppat.1009199.s009.tif]

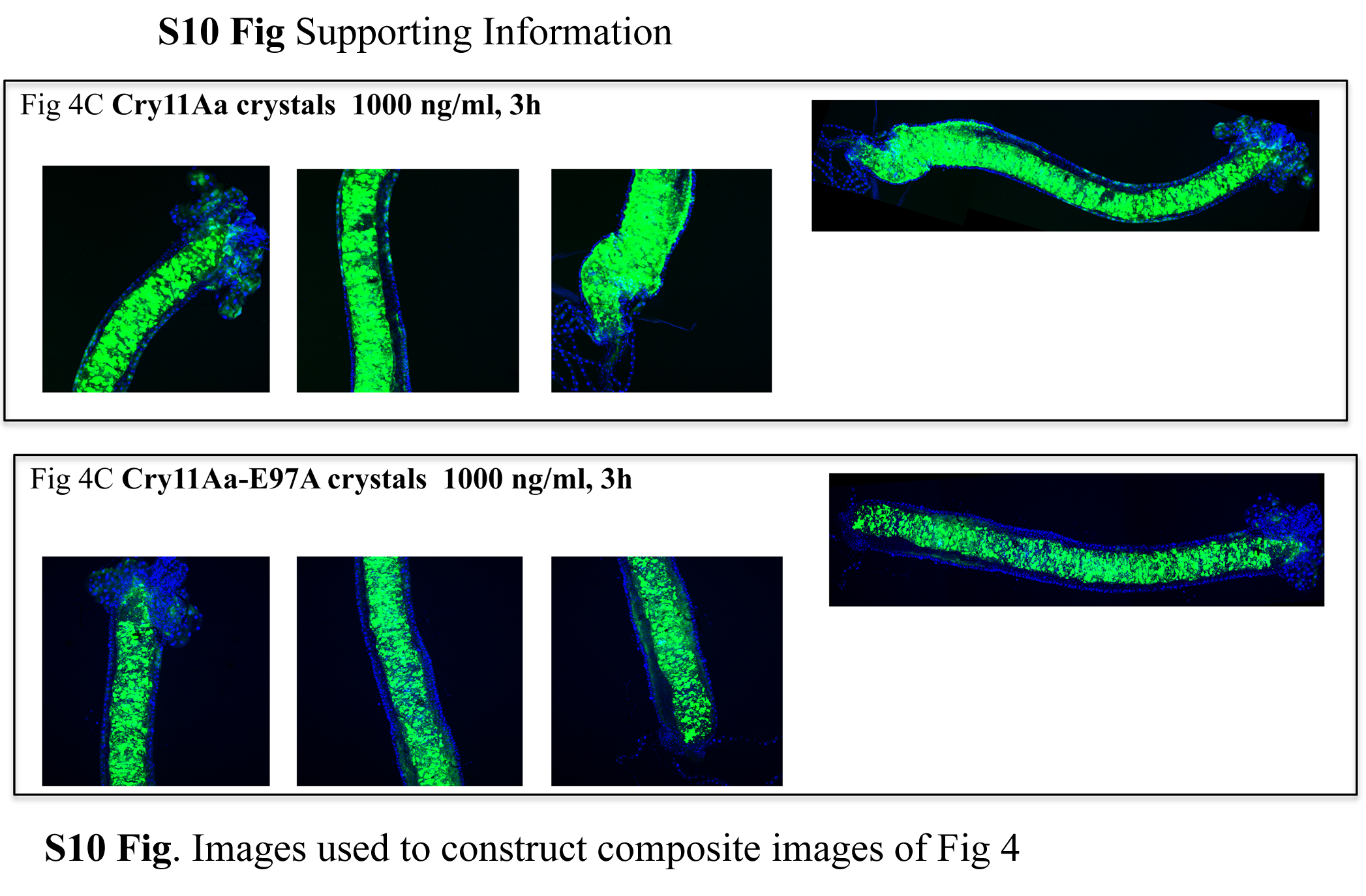

Supplement: S10 Fig — (TIF) [file ppat.1009199.s010.tif]

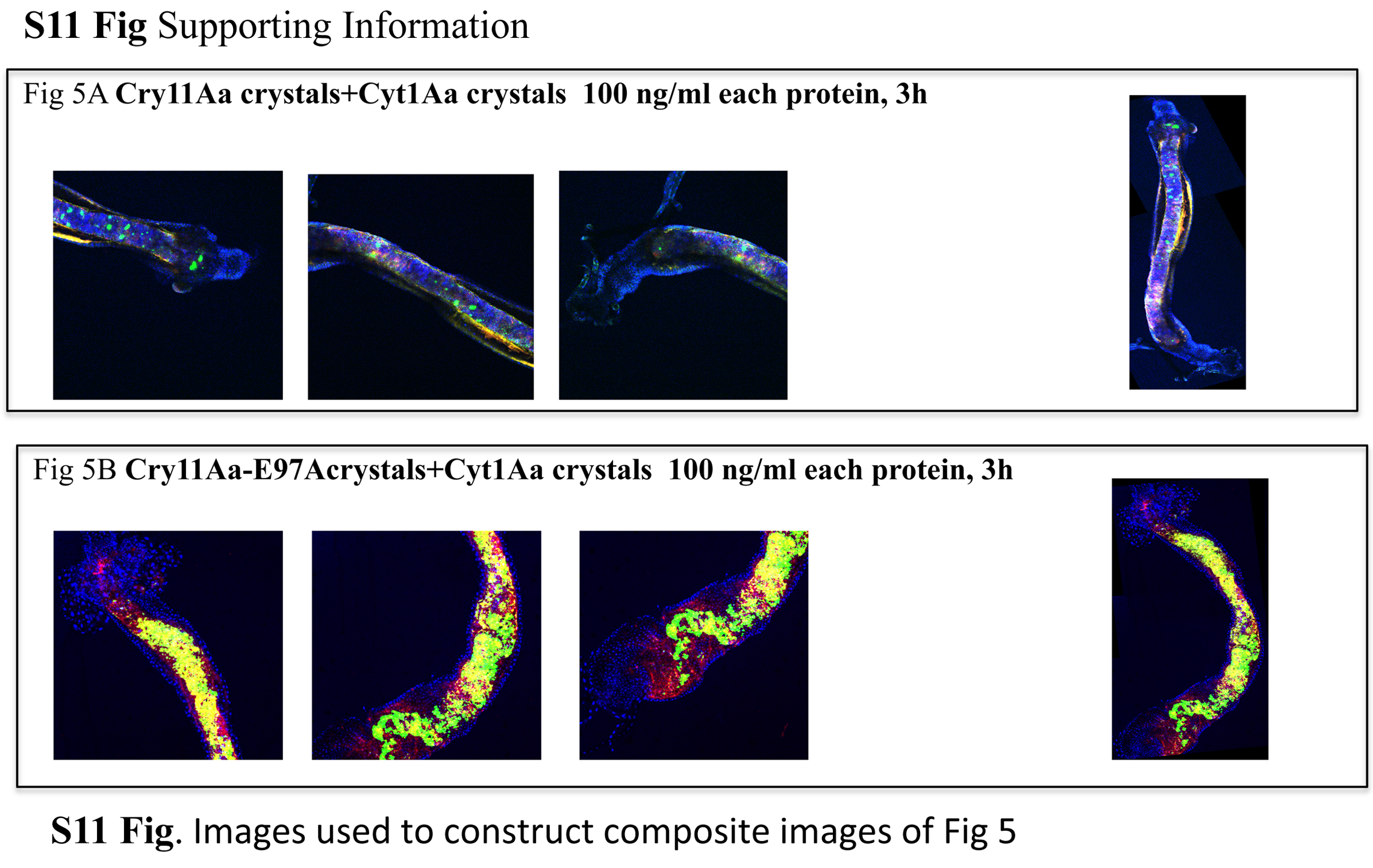

Supplement: S11 Fig — (TIF) [file ppat.1009199.s011.tif]
