# Supplementary material for: In vivo nanoscale analysis of the dynamic synergistic interaction of Bacillus thuringiensis Cry11Aa and Cyt1Aa toxins in Aedes aegypti
Source: PLoS Pathog. 2021 Jan 19;17(1):e1009199. doi: 10.1371/journal.ppat.1009199 (PMC7846010; doi:10.1371/journal.ppat.1009199)
Supplement: Attachment — Submitted filename: PPATHOGENS-D-20-01474.pdf [file ppat.1009199.s012.pdf]

PLOS Pathogens

In vivo nanoscale analysis of the dynamic synergistic interaction of Bacillus thuringiensisCry11Aa and Cyt1Aa toxins in Aedes aegypti.

--Manuscript Draft--

Manuscript Number:	PPATHOGENS-D-20-01474
Full Title:	In vivo nanoscale analysis of the dynamic synergistic interaction of Bacillus thuringiensisCry11Aa and Cyt1Aa toxins in Aedes aegypti.
Short Title:	Nanoscale analysis of Cry11Aa and Cyt1Aa synergism
Article Type:	Research Article
Section/Category:	Bacteriology
Keywords:	Bacillus thuringiensis; Cry toxin; Cyt toxin; synergism; Aedes aegypti; high resolution microscopy; nanoscale
Corresponding Author:	Alejandra Bravo, Ph.D<br>Universidad Nacional Autonoma de Mexico, Instituto de Biotecnologia<br>Cuernavaca, Morelos MEXICO
Corresponding Author's Institution:	Universidad Nacional Autonoma de Mexico, Instituto de Biotecnologia
First Author:	Alejandra Bravo, Ph.D
Order of Authors:	Alejandra Bravo, Ph.D
	Samira López-Molina
	Nathaly Alexandre do Nascimento
	Maria Helena Neves Lobo Silva-Filha
	Adán Guerrero
	Jorge Sánchez
	Sabino Pacheco
	Sarjeet Gill
	Mario Soberón
Abstract:	Bacillus thuringiensis produce crystal inclusions composed of insecticidal proteins, such as Cry11Aa and Cyt1Aa,which work synergistically inducing high toxicity against mosquito larvae. It was proposed that crystal inclusions are rapidly solubilized and activated in the gut lumen, followed by pore formation in the midgut cells killing the larvae. The activated Cry11Aa binds to ALP and cadherin receptors located in gastric caeca and posterior regions, while Cyt1Aa binds to membrane lipids of all midgut regions. Cyt1Aa functions as a Cry11Aa binding receptor, inducing Cry11Aa oligomerization and membrane insertion. Here, we used fluorescent labeled crystals, protoxins or activated toxins for in vivo localization at nano-scale resolution. We show that soluble proteins were not toxic since larvae stop feeding due to their potential toxic activity, since larvae readily ingested soluble non-toxic mutant proteins. Only crystal inclusions were toxic, which remain insoluble in the gut-lumen for a prolonged time. At macroscopic level, Cry11Aa completely degraded the gastric caeca structure and, in the presence of Cyt1Aa, this effect was observed at lower toxin concentrations and shorter period. The labeled Cry11Aa crystal, after midgut processing, binds to the gastric caeca and posterior midgut regions, but also to anterior and medium regions where it is internalized in ordered “net like” structures, leading finally to cell break down. During synergism both Cry11Aa and Cyt1Aa toxins showed a dynamic layered array at the surface of apical microvilli, where Cry11Aa is localized in the lower layer closer to the cell cytoplasm, and Cyt1Aa is layered over Cry11Aa. This array depends on the pore formation activity of Cry11Aa, since the non-toxic mutant Cry11Aa-E97A, unable to oligomerize, inverted this array. Internalization of Cry11Aa was also observed during synergism. These data indicate thatmechanism of action of Cry11Aa is more complex than previously anticipated, and may involve additional steps besides

Powered by Editorial Manager® and ProduXion Manager® from Aries Systems Corporation

	pore-formation activity.
**Suggested Reviewers:**	Juan Luis Jurat-Fuentes<br>University of Tennessee<br>jurat@utk.edu<br>Expert in the study of the mechanism of action of Cry toxins from Bacillus thuringiensis
	Humberto Lanz<br>Instituto Nacional de Salud Publica<br>humberto@insp.mx<br>Expert in Mosquito physiology
	Arieh Zaritsky<br>Ben-Gurion University of the Negev<br>ariehz@bgu.ac.il<br>Expert in the mechanism of action of Cry and Cyt toxins against mosquitoes. He described the structure of Cyt1Aa protein
	David G. Heckel<br>Max-Planck-Institut fur chemische Okologie<br>heckel@ice.mpg.de<br>Expert in the study of the mechanism of action of insecticidal proteins
	Michael J. Adang<br>Georgia Central University<br>adang@uga.edu<br>Expert in the study of Cry and Cyt toxins from Bt. He was one of the first researcher to localize binding of these toxin to midgut tissue sections
**Opposed Reviewers:**	
**Additional Information:**	
**Question**	**Response**
**Financial Disclosure**<br><br>Enter a financial disclosure statement that describes the sources of funding for the work included in this submission. Review the submission guidelines for detailed requirements. View published research articles from *PLOS Pathogens* for specific examples.<br><br>This statement is required for submission and **will appear in the published article** if the submission is accepted. Please make sure it is accurate.	This work was supported by Dirección General de Asuntos del Personal Académico de la Universidad Nacional Autónoma de México-DGAPA/UNAM IN202718 (SP) and DGAPA/UNAM IN203619 (AB), National Institutes of Health-NIH 2R01 AI066014 (SSG, AB and MS). The funders had no role in study design, data collection and analysis, decision to publish, or preparation of the manuscript.

Powered by Editorial Manager® and ProduXion Manager® from Aries Systems Corporation

**Unfunded studies**<br>Enter: *The author(s) received no specific funding for this work.*<br><br>**Funded studies**<br><br>Enter a statement with the following details:<br>• Initials of the authors who received each award<br>• Grant numbers awarded to each author<br>• The full name of each funder<br>• URL of each funder website<br>• Did the sponsors or funders play any role in the study design, data collection and analysis, decision to publish, or preparation of the manuscript?<br>• **NO** - Include this sentence at the end of your statement: *The funders had no role in study design, data collection and analysis, decision to publish, or preparation of the manuscript.*<br>• **YES** - Specify the role(s) played.<br><br>* typeset	
**Competing Interests**<br><br>Use the instructions below to enter a competing interest statement for this submission. On behalf of all authors, disclose any competing interests that could be perceived to bias this work—acknowledging all financial support and any other relevant financial or non-financial competing interests.<br><br>This statement **will appear in the published article** if the submission is accepted. Please make sure it is accurate. View published research articles from *PLOS Pathogens* for specific examples.	No authors have competing interest.<br>The authors have declared that no competing interest exist in this work.

**NO authors have competing interests**<br><br>Enter: *The authors have declared that no competing interests exist.*<br><br>**Authors with competing interests**<br><br>Enter competing interest details beginning with this statement:<br><br>*I have read the journal's policy and the authors of this manuscript have the following competing interests: [insert competing interests here]*<br><br>* typeset	
**Data Availability**<br><br>Authors are required to make all data underlying the findings described fully available, without restriction, and from the time of publication. PLOS allows rare exceptions to address legal and ethical concerns. See the PLOS Data Policy and FAQ for detailed information.<br><br>A Data Availability Statement describing where the data can be found is required at submission. Your answers to this question constitute the Data Availability Statement and **will be published in the article**, if accepted.<br><br>**Important:** Stating 'data available on request from the author' is not sufficient. If your data are only available upon request, select 'No' for the first question and explain your exceptional situation in the text box.<br><br>Do the authors confirm that all data underlying the findings described in their manuscript are fully available without restriction?	Yes - all data are fully available without restriction
**Describe where the data may be found in full sentences. If you are copying our sample text, replace any instances of XXX with the appropriate details.**<br>• If the data are **held or will be held in a**	All relevant data are within the manuscript and its Supporting Information files.

**public repository**, include URLs, accession numbers or DOIs. If this information will only be available after acceptance, indicate this by ticking the box below. For example: *All XXX files are available from the XXX database (accession number(s) XXX, XXX.*). • If the data are all contained **within the manuscript and/or Supporting Information files**, enter the following: *All relevant data are within the manuscript and its Supporting Information files.* • If neither of these applies but you are able to provide **details of access elsewhere**, with or without limitations, please do so. For example: <br>*Data cannot be shared publicly because of [XXX]. Data are available from the XXX Institutional Data Access / Ethics Committee (contact via XXX) for researchers who meet the criteria for access to confidential data.* <br>*The data underlying the results presented in the study are available from (include the name of the third party and contact information or URL).* • This text is appropriate if the data are owned by a third party and authors do not have permission to share the data. <br>* typeset	
Additional data availability information:	

Powered by Editorial Manager® and ProduXion Manager® from Aries Systems Corporation

Dear Sirs, 6 July, 2020

Enclosed you will find the manuscript entitled: "*In vivo* Nano scale analysis of the dynamic synergistic interaction of *Bacillus thuringiensis* Cry11Aa and Cyt1Aa toxins in *Aedes aegypti*" that we submit for consideration to be published in Plos Pathogens.

Bacillus thuringiensis subs *israelensis* produce different pore-forming toxins with distinct modes of action that together show high insecticidal activity against mosquitoes. Among these toxins, Cyt1Aa plays a mayor role in killing mosquitoes since it is able to synergize the insecticidal activity of Cry11Aa, functioning as a Cry11Aa binding receptor, inducing its oligomerization and membrane insertion. *In vivo* interaction of Cry11Aa and Cyt1Aa proteins was never studied, until now. Here we analyzed their *in vivo* interaction at nanoscale resolution. Our results show that they display a dynamic and highly ordered array in the apical microvilli of midgut cells.

This manuscript breaks down some paradigms that were proposed before to explain the mode of action of these toxins. First, the soluble proteins were not toxic to the larvae because the larva stops feeding. Our data indicate that crystal inclusions may mask the toxicity of the insecticidal proteins allowing ingestion by the larvae of high quantities of Bt proteins, and avoiding a potential anti-feeding behavior. Second, we observed internalization of Cry11Aa in ordered "net like" structures within midgut cells and inducing destruction of midgut structures, indicating that the mode of action of Cry11Aa is more complex than previously proposed, involving other effects besides pore-formation. Finally, our high-resolution microscopy analyses revealed that Cyt1Aa and Cry11Aa have a dynamic ordered layered organization pattern in the membrane microvilli, which is compatible with the mechanism where Cyt1Aa functions as a receptor of Cry11Aa facilitating its oligomerization, and inducing its membrane insertion.

We hope that you find this manuscript suitable for publication in Plos Pathogens.

Potential reviewer could be:
Dr. Juan Luis Jurat-Fuentes, University of Tennessee, jurat@utk.edu
Dr. Jeff Fabrick, USDA ARS, jeff.fabrick@ars.usda.gov
Dr. David G. Heckel, Max Planck Institute for chemical Ecology, heckel@ice.mpg.de
Michael J. Adang, University of Georgia, adang@uga.edu

As opposed reviewers, due to conflict of interests we kindly ask please do not send this manuscript to Dr. Lee A. Bulla, Jr, Dr. Jean Louis Schwartz, Dr. Reynald Laprade, or Dr Donald Dean.

Sincerely

Dr Alejandra Bravo
Corresponding author
Instituto de Biotecnologia UNAM

Manuscript
Click here to access/download;Manuscript;Samira2020_FINAL.docx

In vivo nanoscale analysis of the dynamic synergistic interaction of *Bacillus thuringiensis* Cry11Aa and Cyt1Aa toxins in *Aedes aegypti*.

Samira López-Molina[a], Nathaly Alexandre do Nascimento[b], Maria Helena Neves Lobo Silva-Filha[b], Adán Guerrero[c], Jorge Sánchez[a], Sabino Pacheco[a], Sarjeet Gill[d], Mario Soberón[a], Alejandra Bravo[a*]

[a]*Departamento de Microbiología Molecular, Instituto de Biotecnología, Universidad Nacional Autónoma de México (UNAM). Cuernavaca, Morelos, 62250 Mexico.* [b]*Department of Entomology, Instituto Aggeu Magalhães-FIOCRUZ, Recife-PE* 50740-465, *Brazil.* [c]*Laboratorio Nacional de Microscopía Avanzada, Instituto de Biotecnología, Universidad Nacional Autónoma de México (UNAM), Cuernavaca, Morelos 62210, Mexico.* [d]*Department of Molecular, Cell and Systems Biology, University of California, Riverside, Riverside, CA 92521, U.S.A.*

***Corresponding author**: Email address: bravo@ibt.unam.mx

Running title: Nano-scale analysis of Cry11Aa and Cyt1Aa synergism

Keywords: *Bacillus thuringiensis*, Cry toxin, Cyt toxin, synergism, *Aedes aegypti*, high resolution miroscopy

Abstract

Bacillus thuringiensis produce crystal inclusions composed of insecticidal proteins, such as Cry11Aa and Cyt1Aa, which work synergistically inducing high toxicity against

mosquito larvae. It was proposed that crystal inclusions are rapidly solubilized and activated in the gut lumen, followed by pore formation in the midgut cells killing the larvae. The activated Cry11Aa binds to ALP and cadherin receptors located in gastric *caeca* and posterior regions, while Cyt1Aa binds to membrane lipids of all midgut regions. Cyt1Aa functions as a Cry11Aa binding receptor, inducing Cry11Aa oligomerization and membrane insertion. Here, we used fluorescent labeled crystals, protoxins or activated toxins for *in vivo* localization at nano-scale resolution. We show that soluble proteins were not toxic since larvae stop feeding due to their potential toxic activity, since larvae readily ingested soluble non-toxic mutant proteins. Only crystal inclusions were toxic, which remain insoluble in the gut-lumen for a prolonged time. At macroscopic level, Cry11Aa completely degraded the gastric *caeca* structure and, in the presence of Cyt1Aa, this effect was observed at lower toxin concentrations and shorter period. The labeled Cry11Aa crystal, after midgut processing, binds to the gastric *caeca* and posterior midgut regions, but also to anterior and medium regions where it is internalized in ordered "net like" structures, leading finally to cell break down. During synergism both Cry11Aa and Cyt1Aa toxins showed a dynamic layered array at the surface of apical microvilli, where Cry11Aa is localized in the lower layer closer to the cell cytoplasm, and Cyt1Aa is layered over Cry11Aa. This array depends on the pore formation activity of Cry11Aa, since the non-toxic mutant Cry11Aa-E97A, unable to oligomerize, inverted this array. Internalization of Cry11Aa was also observed during synergism. These data indicate that mechanism of action of Cry11Aa is more complex than previously anticipated, and may involve additional steps besides pore-formation activity.

Author Summary

It was proposed that Cyt1Aa functions as a receptor of Cry11Aa, explaining their synergism that increases toxicity against mosquitos. *In vivo* interaction of Cry11Aa and Cyt1Aa proteins has never been studied. Here, we describe the effects of both toxins on gut structures, their localization and action after ingestion. We use quantitative super resolution imaging to analyze their interaction, showing a dynamic and highly ordered array of these proteins in the apical microvilli of midgut cells and internalization of Cry11Aa upon pore formation activity. Our data indicate that the mechanism of action of these pore-forming toxins differs, since Cyt1Aa exerts its activity on the plasma membrane, while additional intracellular effectors are likely to be involved in toxicity of Cry11Aa toxin.

Introduction

Bacterial pathogens make use of different and diverse virulent factors to infect their hosts, among them the pore forming toxins represent highly efficient tools to disrupt target cells. *Bacillus thuringiensis* (Bt) bacteria are insect pathogens that produce, among other virulence factors, different pore forming toxins to break down the midgut epithelial cells of their target insects [1]. During the sporulation phase of growth, different Bt strains produce diverse δ-endotoxins that are concentrated in the mother cell as crystal inclusions [1, 2]. The insecticidal δ-endotoxin proteins characterized so far, are pore forming toxins named Cry or Cyt proteins. The Cry proteins consist of at least three different non-related phylogenetically families showing different primary sequences and three-dimensional structures. These families are named the three-domain Cry toxins (3d-Cry), the Cry-Bin like, and the Cry-Mtx-like toxins [1, 2].

Most Bt strains that kill mosquitoes have the particularity to produce more than one Cry and Cyt toxins that, together, contribute to the toxicity against the target insect pest [1, 2]. One case is the Bt var *israelensis* (Bti) that produce at least three 3d-Cry toxins, such as Cry4Aa, Cry4Ba, and Cry11Aa and one major Cyt toxin, the Cyt1Aa [3, 4]. Bti has a spectrum of action against dipteran insects including *Aedes aegypti, Anopheles gambiae* and *Culex pipiens* mosquitoes, which are vectors of several important human diseases such as dengue, Zika, chikungunya, malaria, encephalitis and filariasis [3, 4]. Both, Cry and Cyt proteins, are produced as protoxins that accumulated in crystal inclusions during Bt bacteria sporulation. When the larvae ingest the crystals, they are solubilized in the alkaline and reducing conditions of the gut and these soluble protoxins are then activated by midgut proteases [1, 2, 4]. Specifically, the Cry11Aa protoxin of 70 kDa is activated to two fragments of 32 and 36 kDa that remain associated as a single ≈65 kDa activated toxin. The 3d-Cry activated toxins are composed of three domains, where domain I is a seven α-helix bundle involved in toxin oligomerization, membrane insertion and pore-formation; while domains II and III, are mainly composed of β-strands, which are involved in the specific interaction with protein receptors and thus directly participate in toxin specificity [1, 2, 4]. In contrast, the activated Cyt toxin has a size of 22 kDa, and it is composed of a single α–β domain, where the two outer layers of α-helix hairpins wrap around a long β-sheet [4-7].

The mosquitocidal 3d-Cry toxins rely on binding to different larval midgut proteins such as alkaline phosphatase (ALP), aminopeptidase-N (APN) or cadherin (CAD) for their membrane insertion and pore formation. These host proteins that function as toxin receptors trigger oligomerization and membrane insertion of the toxin, leading to pore formation and

cell burst is driven by osmotic shock [1, 2]. In contrast, the Cyt toxin does not rely on binding to gut protein receptors, but it specifically binds to lipid membranes triggering also toxin oligomerization, and pore formation after insertion into the membrane [1, 8].

Bti has been widely used for the control of different dipteran pests in the field for more than forty years, without evidence of resistance evolution by the target insects; neither has been possible to artificially select resistant mosquitoes to Bti crystal inclusions under laboratory conditions [9]. Interestingly, the lack of resistance toward Bti crystals was shown to be mainly due to the presence of the Cyt1Aa protein. Resistant insects towards the three individual 3d-Cry toxins present in this strain have been selected in laboratory conditions, but no resistant population could be selected against Cyt1Aa protein [9]. In addition, it was shown that in the presence of Cyt1Aa, the Cry-resistant larvae recovered susceptibility to these Cry4Aa, Cry4Ba and Cry11Aa proteins [10]. Moreover, Cyt1Aa synergize the toxicity of Cry11Aa and Cry4Ba to different mosquito larvae, acting as their receptor, which greatly reduces the selection of resistance [11-15]. It was shown that Cyt1Aa and Cry11Aa proteins bind with each other in a high affinity interaction, that directly correlates with their synergism, as demonstrated by point mutations in the specific regions of Cry11Aa and Cyt1Aa that participate in their binding interaction [12]. Specifically, the exposed domain II loop regions of both Cry11Aa and Cry4Ba toxins, which are involved in the interaction with larval gut ALP receptor, also contributes to their binding to Cyt1Aa [12, 14]. Furthermore, it was also shown that Cyt1Aa induce oligomerization of Cry11Aa resulting in pore formation in membrane vesicles [13], supporting that Cyt1Aa functions as a receptor of Cry11Aa and Cry4Ba proteins, hence explaining its capacity to counter resistance to Cry toxins and to synergize their toxicity against mosquitoes [12-14]. Despite the fact that the mode of action and the synergistic

mechanism between Cyt1Aa and Cry11Aa has been described in some detail, their localization inside the gut after ingestion and effects of both toxins on the midgut cells of the gut structures have not been studied. Immunocytochemical localization of the individual toxins of Bti, after *in vitro* binding to midgut tissue isolated from *An. gambiae* or *Ae. aegypti* larvae, showed that Cry11Aa mainly binds to brush border membrane (BBM) of the gastric *caeca* and posterior midgut regions, while Cyt1Aa binds to BBM of *caeca*, anterior and posterior midgut [16, 17]. The binding of Cry11Aa to the gastric *caeca* and to the posterior midgut correlates with the localization of ALP, APN and CAD receptors in these midgut regions [17-20]. Nevertheless, the interaction of Cry11Aa and Cyt1Aa proteins has never been studied *in vivo*.

Results

Comparative activity of Cry11Aa and Cyt1Aa proteins against *Aedes aegypti* larvae

It was previously reported that soluble Cry11Aa or Cyt1Aa proteins were not toxic to *Ae. aegypti* larvae, in contrast to their crystal inclusions that showed high toxicity [21, 22]. This observation is reinforced by the fact that mosquito larvae are filter feeders that selectively retain particles while soluble material and water are excluded from the gut [21, 22].

We compared the toxicity of crystal inclusions and soluble proteins of Cry11Aa and Cyt1Aa. As controls we used two non-toxic mutants: Cry11Aa-E97A and Cyt1Aa-V122E. The former protein contains a mutation at domain I of Cry11Aa, and the later contains a mutation within helix α-3 of Cyt1Aa. Both mutants exhibit very low toxicity because they were affected in oligomerization [23, 24]. Figure 1 shows that Cry11Aa was only toxic to

larvae when fed as crystal inclusions (LC_{50}= 329.3 ng/ml, confident intervals (CI): 203.1-605.6). The crystal inclusions of the negative control Cry11Aa-E97A protein did not show any toxicity even at 10,000 ng/ml, as previously reported [23]. The soluble Cry11Aa protoxin or activated toxin showed only negligible activity at 10,000 ng/ml (Fig. 1A). In the case of Cyt1Aa, similar results were recorded, Cyt1Aa crystal inclusions showed toxicity to the larvae (LC_{50}= 679.23 ng/ml, CI: 554.9-851.1), while crystals from the negative control Cyt1Aa-V122E mutant showed negligible toxicity, as expected (Fig. 1B) [24]. The soluble Cyt1Aa activated toxin or protoxin showed negligible activity at the highest concentration, 10,000 ng/ml (Fig. 1B).

We also analyzed the toxicity of a 1:1 mixture of Cry11Aa with Cyt1Aa proteins using crystal inclusions or soluble protoxins. The mixture of Cyt1Aa and Cry11Aa crystal inclusions showed a synergism factor (SF) of 8.6 (LC_{50}=51.53 ng/ml, CI: 554.9-851) according to Tabashnik's equation [25] (Fig. 1C). In contrast, the mixtures of soluble Cry11Aa and Cyt1Aa activated toxins did not show toxicity to the larvae (Fig. 1C), while the mixture of soluble protoxins showed one hundred-fold less toxicity compared to the mixture of crystal inclusions (LC_{50}=5335 ng/ml, CI: 4503-6339) (Fig. 1C).

Gut integrity is severely affected after intoxication with Cry11Aa or with a mixture of Cry11Aa and Cyt1Aa proteins

To determine the effect of Cry11Aa and Cyt1Aa proteins on the larval gut structure, we fed 4th instar larvae with crystal inclusions of Cry11Aa, Cyt1Aa or a mixture of crystals from both proteins for different times, and their dissected guts were analyzed by optical microscopy.

Representative images of larval guts from non treated larvae (Fig. 2A) compared with Cry11Aa crystals treatment clearly showed that, after 24 h of a high dose (1000 ng/ml) ingestion, the gastric *caeca* region was completely degraded (Fig. 2B, see blue arrow). In contrast, larvae fed with Cyt1Aa crystals at that same concentration and exposure time showed no evident changes on the *caeca*, or any other gut structure at the macroscopic level (Fig. 2C), even though the tested protein concentration was over the LC_{50} value and that half of those larvae died after 24 h of toxin exposure.

When larvae were fed with a mixture of Cry11Aa and Cyt1Aa crystals at ten times lower concentration (100 ng/ml Cry11Aa + 100 ng/ml Cyt1Aa) the *caeca* gut region was completely degraded after only 3 h (Fig 2D, see blue arrow), which could be due to the enhanced mortality observed in the bioassays performed with a mixture of both proteins (Fig. 1C). Larvae fed with a mixture of crystals from the nontoxic mutant Cry11Aa-E97A with wild type Cyt1Aa (1000 ng/ml Cry11Aa-E97A + 500 ng/ml Cyt1Aa) showed an intact *caeca* gut region after 24 h (Fig. 2E), suggesting that degradation of the *caeca* in the mixture of toxins may be due to enhanced Cry11Aa toxicity induced by Cyt1Aa.

Larvae did not ingest either soluble protoxin or activated Cry11Aa toxin.

To determine the fate of Cry11Aa or Cyt1Aa in the gut tissue of larvae fed with these proteins. Cry11Aa or Cry11Aa-E97A crystals or soluble proteins were labeled with Alexa-546 (green color), while Cyt1Aa or CytAa-V122E crystals or soluble proteins were labeled with Alexa-647 (red color) (Fig. S1). The labeled crystals showed similar toxicity to the wild type protein, which was verified by bioassays prior to their use (data not shown). The efficiency of labeling of each protein sample was determined with respect to its molar extinction coefficient as described in materials and methods and labeled proteins

were visualized directly on the SDS-PAGE excited with Epi-RGB trans-illuminator (Fig S1).

When larvae were fed with the activated Cry11Aa-Alexa546 soluble toxin for 24 h, this protein was not observed inside the gut lumen, neither associated to BBM from all midgut regions (Fig. 3A). Only after 3 h of exposure, a discrete detection of soluble toxin was recorded associated to the microvilli membrane of the posterior midgut (Fig S2A, see red arrowhead). However, this localization of the labeled toxin was not further observed at other intoxication times, i.e. after 6 h or longer periods of larval exposure to the toxin (Fig. S2A), supporting that larvae did not ingest the soluble toxin.

When larvae were fed with Cry11Aa-Alexa546 soluble protoxins for 24 h, or shorter incubation times, this protoxin was also barely observed in the gut lumen (Fig 3B and Fig S3A). After 9 h and 24 h of ingestion, some Cry11Aa-Alexa546 protoxin was found internalized in small vesicles into the cells of medium midgut region (Fig. 3B and Fig. S3A, see the orange arrowheads, in the insert images of these figures).

The soluble activated toxin or protoxin from the nontoxic mutant Cry11Aa-E97A were readily ingested by the larvae

In stark contrast, at all times analyzed, soluble activated toxin of the nontoxic mutant Cry11Aa-E97A-Alexa546 accumulated in the gut lumen, and it was found principally associated to the microvilli membranes in the gastric *caeca* and posterior midgut (Fig. 3A and Fig. S2B). This protein was also observed slightly bound to microvilli membrane from anterior and medium region (Fig. 3A and Fig S2B, see white arrowhead in the insert-1 of Fig. 3A). It is important to mention that the mutation of Cry11Aa-E97A is located in helix α-3 of domain I that is involved in oligomerization and pore formation

activity, while the regions of this toxin that are involved in toxin binding are located in domain II and III, implying that binding interaction of this toxin may not be affected [23].

Similarly, at all times analyzed, the soluble protoxin from the non-toxic Cry11Aa-E97A-Alexa546 mutant was observed inside the gut lumen, and also associated with microvilli membrane, principally from the gastric *caeca* and posterior midgut regions (Fig. 3B and Fig. S3B).

These data indicate that the lack of toxicity of Cry11Aa soluble proteins correlated with a strong reduction of their ingestion. This probably occurred due to the toxic nature of these proteins, since the soluble proteins (activated toxin or protoxin forms) from the non-toxic mutant Cry11Aa-E97A-Alexa546 were readily ingested by the larvae, accumulated in the gut lumen and were found are bound to microvilli membranes.

Crystal inclusions from Cry11Aa or Cry11Aa-E97A are ingest by the larvae and accumulated in the gut

Previously, it was shown that when *Ae. aegypti* larvae were fed with low doses of the Cry11Aa crystals, some regions of the microvilli were detached from the cells, and this shedding of membranes was proposed to be a defense mechanism against Cry toxin action [20]. Here we observed a similar behavior, since at low dose (100 ng/ml) ingestion of Cry11Aa-Alexa546 crystals, these inclusion bodies were readily ingested and accumulated into the midgut lumen and the labeled protein was found bound to the *caeca* and the posterior part of the midgut (Fig 3C), but the microvilli membrane from the posterior region was deformed and membrane shedding was also observed (see insert-4 in Fig 3C), as previously reported [20]. However, we also observed that Cry11Aa-Alexa546 was internalized in some cells of the medium midgut and in the *caeca* (Fig 3C, see yellow

arrowhead and insert-3). Cell internalization was observed after 9 h of intoxication with a low dose (100 ng/ml) of Cry11Aa-Alexa546 crystals per ml (Fig 4A, see yellow arrowhead). Larvae intoxicated with a high dose of Cry11Aa-Alexa546 crystals (1000 ng/ml) showed that all cells from their gut tissue were severely damaged after 24 h (Fig. 3C, see red arrow heads and insert-5). At 3 h and 6 h incubation with a high dose (1000 ng/ml) of Cry11Aa-Alexa546 crystals, the labeled protein was found associated with membranes from all midgut regions including anterior and medium midgut (Fig. 4A) and the internalization of this protein in certain cells of the gut is more evident after 3 h and 6 h (Fig. 4A, see yellow arrows). After 9 h, cells from all gut regions were completely broken down (Fig 4A, see red arrows), and the macroscopic structure of the gastric *caeca* was clearly damaged (Fig 4A, see blue arrowhead). Midgut cells that only show toxin bound to the microvilli were named Cry11Aa-P1 and those cells where Cry11Aa-Alexa546 was internalized, showing a strong fluorescence signal were named Cry11Aa-P2 (Fig. 4C, yellow arrow heads). It is possible that in Cry11Aa-P2 cells, the apical membrane may already be permeable, allowing Cry11Aa internalization.

In contrast, when larvae were fed with crystals of the mutant protein Cry11Aa-E97A-Alexa546 at high dose (1000 ng/ml) for 24 h, this labeled protein was found in the midgut lumen and also associated to the microvilli membranes, principally from gastric *caeca* and posterior midgut, but no apparent cell damage or cell internalization was observed (Fig. 3C). Similar images were observed at shorter incubation times (3, 6 and 9 h) (Fig 4B). Slight binding of Cry11Aa-E97A-Alexa546 with microvilli membrane of the anterior and medium midgut regions was also observed (Fig 3C and 4B).

The intensity of fluorescence (IF) of the cytoplasm region was analyzed in Cry11Aa-P1 and Cry11Aa-P2 cells, and compared with Cry11Aa-E97A. Figure 4C shows

a fragment of the midgut tissue that was dissected from an *Ae. aegypti* larvae intoxicated for 3 h with Cry11Aa-Alexa546 or with Cry11Aa-E97A-Alexa546 crystal inclusions. The cytoplasmic IF was measured in tens of individual cells, including the highly brilliant Cry11Aa-P2 (Fig 4C, see yellow arrows), the dark cells Cry11Aa-P1, and cells from the larvae intoxicated with the mutant Cry11Aa-E97A-Alexa546 protein (Fig. 4C). These IF measurements showed that Cry11Aa-P2 cells were significantly brighter than Cry11Aa-P1, or Cry11Aa-E97A-Alexa546 cells (*P* value < 0.001, multiple comparison after Kruskal-Wallis test (Fig. 4C).

During all times of intoxication, the Cry11Aa labeled crystals were observed inside the gut lumen of the larvae (Fig. 4). In order to determine how long these crystals may reside in the gut lumen, larvae were fed for 2 h with 1000 ng/ml of Cry11Aa-Alexa546 crystals and then transferred to clean water and analyzed at different times for the presence of labeled crystals in their gut lumen. Figure S4 shows that after 2, 5 and 7 h of transferring the larvae to clean water, the larval gut lumen still contained Cry11Aa-Alexa546 labeled crystals, indicating that *in vivo* the crystal inclusions may spend a long period of time inside the larval gut.

Soluble Cyt1Aa protoxin also induced cessation of ingestion and only crystal-inclusions of Cyt1Aa were readily ingested by the larvae

Regarding to Cyt1Aa, the interruption of larval ingestion was also observed with the Cyt1Aa-Alexa647 soluble protoxin, since it did not accumulated in the gut lumen and was not associated with membranes from any gut regions at all times analyzed including after 24 h (Fig. 3D, and Fig. S5A). In contrast, the soluble protoxin from nontoxic mutant Cyt1Aa-V122E-Alexa647 was readily ingested and accumulated in the gut lumen (Fig. 3D

and S5B). In the case of larvae intoxicated with Cyt1Aa-Alexa647 crystals, confocal microscopy revealed that labeled crystal were highly accumulated in the gut lumen and Cyt1Aa-Alexa647 was associated with BBM from all gut regions, although no clear cell damage was observed (Fig. 3E and Fig. S6A). Similarly, when larvae were fed with crystals of the non-toxic mutant Cyt1Aa-V122E-Alexa647 strong accumulation of this protein was observed in the gut lumen and was found associated with BBM from all gut regions (Fig. 3E and Fig. S6B). These data confirm that the lack of toxicity of Cyt1Aa soluble proteins is also due to a cessation of ingestion by the larvae.

In supplementary figures S2, S3, S5 and S6, bright field images of the gastric *caeca* at all periods of intoxications with the different protein samples (Cry11Aa and Cyt1Aa activated and protoxins) are shown. These images indicate that none of these conditions impair the *caeca* structure; the only exceptions were with Cry11Aa crystals, where the damage of *caeca* structure was evident after 9 and 24 h treatment (Fig. 2B and Fig 4A, see blue arrowheads).

Cry11Aa and Cyt1Aa colocalize during their synergistic interaction in *Aedes aegypti* larval guts

To determine the fate of Cry11Aa and Cyt1Aa proteins during their synergistic interaction in the gut, 4th instar larvae were fed with a mixture of crystal inclusions of Cry11Aa-Alexa546 and Cyt1Aa-Alexa647 (1:1 ratio, at 100 ng/ml concentration of each toxin). After 3 h of ingestion the midgut tissue was dissected and observed under the confocal microscope. Figure 5A shows that, both Cry11Aa-Alexa546 and Cyt1Aa-Alexa647 crystals accumulated in gut lumen. Both toxins were associated and colocalized at microvilli membranes from all gut regions, including the anterior, medium and posterior

midgut regions (Fig. 5A). The *caeca* was already disrupted under these conditions, thus we observed no colocalization in this region. Colocalization of both toxins in the microvilli of gastric *caeca* was clearly recorded when larvae were fed with a mixture of mutant Cry11AaE97A-Alexa546 and Cyt1Aa-Alexa647 (Fig 5B).

During synergism of Cry11Aa and Cyt1Aa proteins, cell internalization of Cry11Aa-Alexa546 toxin was observed in the cells from the medium and posterior parts of the gut (Fig. 5A see yellow arrows in insert-1 and insert-2). The cells from the posterior region were highly affected after 3 h of intoxication, showing awry morphologies (Fig. 5A, see insert-1).

When a mixture of crystals of the mutant Cry11Aa-E97A-Alexa546 and Cyt1Aa-Alexa647 (1000 ng/ml + 500 ng/ml, respectively) was used to feed larvae, both toxins were found to be associated and colocalized bound to microvilli membranes from all gut regions. No internalization of these toxins was evident, neither apparent cell damage was observed at 3 h (Fig 5B and insert-3) nor at 9 h (Fig S7) of ingestion. At such times, microvilli structures are intact and the size of the cells do not change even though that the concentration of labeled proteins used was much higher that at which the wild type toxins were analyzed, as noted above (Fig. 5B and Fig. S7).

IF analysis of the cytoplasm region from tens of cells in each condition confirmed these data, since cells intoxicated simultaneously with Cry11Aa-Alexa546 + Cyt1Aa-Alexa647 have statistical significant higher IF values, compared to cells intoxicated with a mixture of Cry11Aa-E97A-Alexa546 + Cyt1Aa-Alexa647 (Fig. 5C; P value < 0.001, Kruskal-Wallis test).

Cry11Aa and Cyt1Aa proteins arrange into specific nanoscopic patterns at the apical region of the cell and in the cytosol

To address the nanoscopic location of Cry11Aa inside the cells and the observed colocalization of Cry11Aa and Cyt1Aa proteins during their synergistic interaction in the gut, we made use of super resolution radial fluctuation (SRRF) analysis, since this microscopy imaging technique overcomes the diffraction limits of confocal microscopy, providing nanoscopic information of fluorescent labeled structures [26]. First, we analyzed the images of larvae fed with Cry11Aa-Alexa546, crystals. Highly brilliant cells (Cry11Aa-P2) showing internalized Cry11Aa-Alexa546 protein were compared with the dark cells (Cry11Aa-P1), which correspond to cells where the toxin was bound to the microvilli but was not internalized (Fig. 4A). High-resolution analyses revealed that, besides being associated with the apical microvilli (Fig 6A and inserts), Cry11Aa-Alexa546 protein is internalized in structures displaying a "net like" organization within the whole cell cytoplasm (Fig 6A and inserts). In contrast, the non-toxic Cry11Aa-E97A-Alexa546 mutant protein was only associated to the apical membrane (Fig. 6B and inserts).

To determine whether such "net-like" *xy* pattern organization appear to be clustered or randomly distributed, we scrutinized the cellular milieu of tents of cells by means of computing the Ripley's K~ function [27] that computes the average number of particles located within a predefined radius of any typical event and addresses organization on fluorescent images, since it considers fluorescence intensity as a proxy of the event density [27]. Our data revealed a remarkable degree of organization within the cytoplasm of Cry11Aa-P2 cells. The K~ function computed over the Cry11Aa-Alexa546 distribution indicated that this protein accumulated inside the cells in an ordered fashion (Fig 6A insert -2 and Fig. 6D). Such organization was found to be over represented at the submicron scale,

where the K~ statistics peaks. Nonetheless, the observed degree of organization also scales down to the micron regimes (as observed in inserts of Fig. 6D). Such degrees of organization were less prominent in the Cry11Aa-P1 dark cells under the same conditions (Fig. 6A and Fig. 6D), or when the larvae were fed with the mutant Cry11Aa-E97A-Alexa546 (Fig. 6B and Fig. 6E). These observations are strengthened by comparing the distribution of K~ maxima of Cry11Aa-P2, with the corresponding computed values of either Cry11Aa-P1 or Cry11Aa-E97A-Alexa546 protein distributions (*P*-value < 0.05, multiple comparisons after Kruskal-Wallis test) (Fig. 6F).

The high-resolution images of cells intoxicated with Cyt1Aa-Alexa647 showed that this protein was associated with the apical membrane (Fig. 6C) and that its internalization was moderate compared to labeled Cry11Aa (Fig. 6C and Fig. 6G).

Cry11Aa and Cyt1Aa distribution during synergism exhibit a dynamic nanoscopic organization layered at the apical membrane of the cell.

It was shown that the synergism between Cry11Aa and Cyt1Aa toxins involves a high affinity interaction that was demonstrated by *in vitro* binding assays and also that this interaction facilitates Cry11Aa oligomerization, increasing its pore formation activity [12, 13]. Figure 5A shows that Cry11Aa and Cyt1Aa proteins colocalize at the microvilli membranes, hence supporting a putative synergistic interaction that might involve molecular contact as indicated by the binding assays reported before. However, image resolution by confocal microscopy is limited by diffraction, to 0.2 – 0.3 μm. As result of that, the observed colocalization in the confocal microscopy, precludes us to derive conclusions at the nanoscale level.

To determine whether the observed colocalization (Fig 5A) when 4th instar larvae were fed 3 h with a 1:1 mixture of crystals from both proteins (100 ng/ml of each protein) is due to a precise arrange at the apical membrane of the cell, we analyzed their distribution by super resolution SRRF microscopy [26]. Figure 7A show that these proteins are layered into specific foci, positioned on the external region of the apical membrane of the cell. Cyt1Aa-Alexa647 was found in the upper layer, while Cry11Aa-Alexa546 was located down facing the cell cytoplasm (Fig. 7A, see red arrowhead in insert-1). Surprisingly, the analysis of the interaction of the mutant Cry11Aa-E97A-Alexa546 (1000 ng/ml) with Cyt1Aa-Alexa647, (500 ng/ml) after 3 h of intoxication, showed an inverted layered organization on the cell microvilli, where Cry11Aa-E97A-Alexa546 protein was found external to Cyt1Aa-Alexa647 at the apical membrane of the cell (Fig. 7B, see yellow arrowhead in insert-3). These data are highly interesting since Cry11Aa-E97A was previously shown to be unable to oligomerize and insert into liposomes and these data correlated with its severely impairment in insecticidal activity [23].

To further quantitate such degree of organization at the apical membrane of cells from treated larvae, we scrutinized the relative distances of both protein-layered distributions. Our initial approximation was to compute line profiles over each protein distribution at the microvilli, and later to compare their proximity by looking at their local maxima. However, stretching and folding of both protein distributions was observed at the nanoscale regimes, hence precluding us to reach statistical significance. To overcome such limitation, we developed an algorithm that allowed us to quantitate the local distance between both protein layers (as described in materials and methods). The algorithm stems on taking Cyt1Aa-Alexa647 distribution as a reference layer to assess its local degree of

deformation computing its spatial paired correlation function, from dozens of regions located in the cell microvilli.

The data showed that both wild type toxins (Cry11Aa-Alexa546 and Cyt1Aa-Alexa647) were located at an average distance between each other of ≈ 26 nm (Fig. 7C). The average distance between Cry11Aa-E97A-Alexa546 mutant with the Cyt1Aa-Alexa647 protein is also ≈ 26 nm, but in the opposite direction (Fig. 7D). Figure 7E shows the distribution of all computed distances between Cry11Aa-Alexa546 or Cry11Aa-E97A-Alexa546 to Cyt1Aa-Alexa647 (where Cyt1Aa is positioned as the zero reference). Note that the distribution of distances between Cry11Aa-Alexa546 and Cyt1Aa-Alexa647 is shifted to the left side of Cyt1Aa (negative values), but the distribution of distances between Cryt11Aa-E97A-Alexa546 and Cyt1Aa-Alexa647 is shifted to the right side of Cyt1Aa (positive values) (Fig. 7E). Kruskal-Wallis test performed over the distributions of Figure 7E indicate statistical difference (P value < 0.0001). Noteworthy, the distribution of all computed distances between Cry11Aa-Alexa546 or Cry11Aa-E97A-Alexa546 to Cyt1Aa-Alexa647 are rather broad, both covering positive and negative values (Fig. 7E). Hence, indicating that the interaction between these proteins is rather dynamic, where Cry11Aa and Cyt1Aa toxins may have different distances between them. The location of the wild type Cry11Aa protein showed two peaks (Fig. 7E), suggesting that this protein may be found in two different conformations, one colocalizing over the Cyt1Aa, and other under the Cyt1Aa facing the cytoplasm, while the mutant Cry11Aa-E97A that is unable to oligomerize could be found colocalizing with Cyt1Aa in only one conformation on the top of the Cyt1Aa protein.

Our super-resolution studies also show that the wild type Cry11Aa-Alexa546 protein was found inside the cells in a net-like structure (Fig. 7A) during synergism with

Cyt1Aa, similar to the organization pattern observed when larvae were fed only with Cry11Aa-Alexa546 crystals (Fig. 6A). Statistical image analysis of the organization of both toxins showed that wild type Cry11Aa-Alexa546 had much higher $K^{\sim}$ values than the mutant Cry11Aa-E97A-Alexa546 (Fig. 7F), indicating internalization of Cry11Aa-Alexa546 in an ordered close and distal conformations. The Kruskal-Wallis test over the distributions of $K^{\sim}$ maxima computed values from the wild type Cry11Aa-Alexa546 protein against Cry11Aa-E97A-Alexa546 $K^{\sim}$ maxima values, revealed statistically significant differences (P value < 0.05) (Fig. 7F). The data confirms our observation that the mutant is not capable of internalization into the cell cytoplasm, even though the concentration of Cry11Aa-E97A-Alexa546 was 10 times fold higher than Cry11Aa-Alexa546 in these comparisons.

From the confocal images shown in Figure 5B in relation to the interaction of Cyt1Aa-Alexa647 with Cry11Aa-E97A-Alexa546, no apparent internalization of Cyt1Aa-Alexa647 inside cells was observed (Fig. 5B). However, the super resolution images revealed some Cyt1Aa-Alexa647 inside the cell (Fig. 7). The analysis of the distribution of Cyt1Aa-Alexa647 inside the cells during the synergism with both Cry11Aa proteins (wild type or mutant) showed similar patterns (Fig. 7G), and Kruskal-Wallis test confirmed that these patterns have no significant differences.

Discussion

It has been documented that insects are able to regulate their feeding behavior when exposed to toxins [28]. In the case of different Lepidopteran insect species, it has been shown that larvae selectively choose diet that does not contain Bt Cry toxins [29-31]. In

contrast, in the case of mosquito larvae no such behavior has been documented before. Mosquito larvae live in an aquatic environment where they feed by filtration of particles including bacteria, zooplankton and algae; but studies of their feeding behavior and selective capacity of ingestion are limited. Here, we show that toxic soluble pore-forming toxins of Cry11Aa or Cyt1Aa do not display toxicity to the larvae due to a reduced ingestion by *Ae. aegypti* larvae. In contrast, soluble non-toxic mutants of these proteins were ingested by larvae and accumulated into their gut lumen. Interestingly, the larvae readily ingested crystal inclusions of Cry11Aa, Cyt1Aa or combination of both proteins despite their high toxicity, probably because these proteins are enclosed in crystals and they are not toxic until they are solubilized under the alkaline pH of midgut lumen [1, 2], suggesting that crystal inclusions may mask the toxicity of the insecticidal proteins allowing ingestion by the larvae of high quantities of Bt proteins, and avoiding a potential anti-feeding behavior. It was proposed that the transit time of Cry and Cyt crystals along the mosquito larvae gut is from 30–60 min, depending on species [32]. Also, that solubilization and toxin activation is a quite fast process, since these two processes can be performed *in vitro* in short incubation times (from 15 min to 1 h at pH 10.5) [1, 3]. Here, we show that *in vivo* the Cry and Cyt crystals may remain in the mosquito lumen as inclusion bodies for extended periods of time (>7 h). It is possible that a slow solubilization of these proteins allowed the ingestion of substantial amount of toxic crystals before the larvae could sense toxicity and stop feeding. Thus, it is tempting to propose that the production of protoxins as crystal inclusions could have been selected during Bti evolution to avoid feeding rejection behavior by the larvae. This hypothesis remains to be demonstrated.

Both Cry11Aa and Cyt1Aa are recognized as pore forming toxins. Nevertheless, observations made here suggest that both toxins exert toxicity by different mechanisms. It was reported that Cry11Aa toxin associates preferentially to microvilli from *caeca* and posterior midgut, which correlates with the sites where Cry11Aa receptors (ALP, APN and CAD) were shown to be preferentially expressed [17-20]. However, our data show that Cry11Aa or the non-toxic Cry11AaE97A mutant also binds to the microvilli of the anterior and medium midgut regions. Interestingly, it was recently shown that CRISPR-Cas9 edited *Ae. aegypti* larvae expressed the CAD fused to the green fluorescent protein (cadEGFP) principally in the *caeca* and posterior midgut regions, but also a low expression of this protein was observed in anterior-medium midgut region [20]. Thus, CAD could participate in Cry11Aa binding to anterior and medium midgut. In the future, it will be important to determine if additional Cry11Aa-receptor molecules are involved in the binding of Cry11Aa toxin to anterior and medium gut regions and to identify them.

The non-toxic Cry11Aa-E97A mutant used in this work is located in helix α-3 of domain I and it is affected in oligomerization in synthetic membranes, suggesting no pore formation activity. We previously showed by circular dichroism analysis, that this mutant retains the structure of Cry11Aa toxin. However, its binding characteristics to BBM isolated from mosquito larvae were not described before [23]. A similar corresponding mutation was isolated before in the lepidopteran-specific Cry1Ab protein, Cry1Ab-R99E mutant. This mutant was also affected in oligomerization, pore formation and toxicity against *Manduca sexta* larvae, and it was demonstrated that it is still able to bind to its receptors, with similar affinity as Cry1Ab [33]. Here we show that Cry11Aa-E97A mutant, although is not toxic, it is still also able to bind to the membranes of the mosquito larvae.

We show that when larvae were fed with crystals of Cry11Aa-Alexa546 at low doses (100 ng of crystals / ml) the toxin was bound to the microvilli of *caeca* and posterior region. Some microvilli shedding was observed at 24 h, as previously described [20]. It was proposed that a rapid loss of the cell microvilli containing the toxic pores of Cry11Aa could be a defense mechanism of the larvae to prevent further damage [20]. However, here we also observed internalization of Cry11Aa in certain cells of the midgut. At higher dose (1000 ng of crystals/ml) the number of cells that showed internalization of Cry11Aa increased, and at longer incubation times these cells were completely degraded. In contrast, Cyt1Aa showed a strong association with the microvilli from all gut regions, but this protein was not accumulated to high levels inside cells and did not induce a clear destruction of the cells during intoxication. These data are interesting since we have previously reported that in the mosquito *Culex quinquefasciatus* the Cyt1Aa toxin also binds to microvilli from all midgut regions, but it also can be observed inside cells, internalized on small vesicles in the cells of *caeca* and anterior midgut regions, although this pattern requires further analysis [34]. Here we observed similar internalization into small vesicles only when *Ae. aegypti* larvae were fed with Cry11Aa protoxin.

An additional striking difference between both Cry11Aa and Cyt1Aa was the massive degradation of the *caeca* gut structure, induced by Cry11Aa but not by Cyt1Aa, even though both toxins were shown to bind to *caeca* microvilli. The complete destruction of the *caeca* structure by Cry11Aa action was not previously reported, and it was remarkable that when larvae were fed with both proteins, Cry11Aa and Cyt1Aa, this effect was synergized. It is possible that internalization of Cry11Aa into cells is responsible for the degradation of the gut structures. Cry11Aa cell internalization in the *caeca* was more difficult to observe, mainly due to the complex structure of *caeca*, that does not allow clear

transversal sections. Interestingly, we show that Cry11Aa cell internalization was triggered by its pore formation activity, since Cry11Aa-E97A mutant, was not internalized into the cells.

Different Cry toxins have been shown to bind actin, and silencing actin expression in *Ae. aegypti* larvae caused increased toxicity of Cry11Aa, supporting a possible defense mechanism [35-38]. Interestingly, Cry5Ba that is toxic against nematodes, is internalized inside gut cells in small vesicles by RAB-5 endocytosis as a defense mechanism, involving microvilli shedding and destruction of Cry5Ba pores after vesicles fusion with the lysosomes [39]. In our opinion this internalization of Cry5Ba in small vesicles is more similar to the internalization that we observed with the soluble Cry11Aa protoxin at 9 and 24 h of intoxication, probably representing also a defense strategy against protoxin action, since all larvae survived after soluble protoxin treatment. It has been shown that other pore-forming toxins cause release of cell blebs containing cytoplasm material, which may result from the engagement of cell death pathways triggered by pore formation activity [40, 41]. It remains to be determined if the reason for the high Cry11A cell internalization, in contrast to Cyt1Aa, as well as to identify the Cry11Aa internalization pathway.

There are multiple studies showing the histopathological effects of Cry toxins in midgut cell observed by light or transmission electron microscopy in different insect orders including lepidopteran [42-47], coleopteran [48] or dipteran larvae [49-52]. Overall, these reports show in that Cry toxins induced severe vacuolization of the cytoplasm of the midgut cells, as well as loss of microvilli structure, cell lysis and columnar cells fragmentation. It was even proposed that vacuolization resulted from increased Golgi vesicles, up to 4 μm in size [44]. It is possible that the highly ordered “net like” structures in the cytoplasm that we observed here, correspond to these vacuolization events, described so far in other insect

pests after treatment with different Cry toxins. Some authors have immunolocalized the toxin in infected larvae. However, none of them observed Cry toxin internalization into cells, only that the toxin binds to the microvilli membrane [42, 47, 50]. One possible explanation for these different results, is that in the analysis of fixed tissues, requires unmasking antigen treatment, to allow the interaction of the antibodies with their epitopes, implying a trypsin treatment of the tissue sections which highly damage the tissue [42, 47, 50]. Another explanation could be that only part of the toxin is internalized into the cells and the antibodies used to immunolocalize did not recognize the cleaved fragment of the protein. Here, we used proteins labeled with fluorescent dyes that bind to Lys residues, which are found all over the Cry11Aa and Cyt1Aa proteins.

Finally, our high-resolution microscopy analyses revealed that Cyt1Aa and Cry11Aa have a dynamic ordered layered pattern of organization in the membrane microvilli, which is compatible with the mechanism where Cyt1Aa functions as a receptor of Cry11Aa facilitating its oligomerization, and inducing its membrane insertion. Interestingly, high-resolution images revealed that Cry11Aa was finally localized facing cell cytoplasm, below the Cyt1Aa in the microvilli. In contrast, the interaction of Cry11Aa-E97A with Cyt1Aa revealed an inverted organization pattern in the microvilli membrane. It has been shown that after membrane insertion, the Cry1Ab oligomers are mobilized into lipid membrane rafts where the toxin inserts and forms pores [53]. Thus, the different organization in the microvilli membrane of the mutant Cry11Aa-E97A is likely due to its defects in oligomerization and membrane insertion.

Overall, our results allowed us to break down some paradigms that were proposed before to explain the mode of action of these toxins. The mode of action of Cry11Aa and Cyt1Aa greatly differs and we show that the mechanism of Cry11Aa is more complex than

previously proposed, involving other effects besides pore-formation. It would be fundamental to determine the intracellular events that are triggered after toxin cell internalization. Also, to identify the Cry11Aa receptors in anterior and medium regions of the larvae. Decoding the complete mechanism of Cry and Cyt toxins is likely to provide future strategies for their efficient use in pest control, and for the control of insects that develop resistance to these toxins.

Materials and methods

Toxins preparation. Cyt1Aa, Cyt1Aa-V122E, Cry11Aa and Cry11Aa-E97A proteins were produced in the *B. thuringiensis* 407 acrystalliferous strain transformed with the corresponding plasmids with genes that encode each toxin protein such as pWF45-Cyt1Aa or pWF45-Cyt1Aa-V122E [24, 54] and pCG6-Cry11Aa or pCG6-Cry11Aa-E97A [23, 55]. The construction and characterization of the non-toxic mutants (Cyt1Aa-V122E and Cry11Aa-E97A), that were affected in oligomerization and insertion into membrane, was previously described [23, 24]. Bacteria cultures were grown in solid nutrient broth sporulation medium [56] supplemented with erythromycin (10 µg/mL) for 72 h until sporulation (≥80 %), detected in an optical microscope. Spores/crystals were recovered and washed once with acidic distilled water adjusted to pH 4 using 5 M HCl, and then three times with 0.3 M NaCl, 0.01 M EDTA, pH 8.0 and three times with 1 mM phenylmethylsulfonyl fluoride (PMSF) and stored at 4 ºC. Cry11Aa and Cry11Aa-E97A crystal inclusions were purified by sucrose gradient centrifugation [57], while Cyt1Aa and Cyt1Aa-V122E inclusions were purified by the aqueous two-phase system composed of phosphate buffer 40% and polyethylene glycol (PEG) 40% as previously described [22,

58]. Cry11Aa and Cry11Aa-E97A protoxins were solubilized in 50 mM Na_2CO_3, 0.2% β-mercaptoethanol, pH 10.5 and centrifuged for 10 min at 12,850 × *g*, 4 ˚C. Cyt1Aa and Cyt1Aa-V122E were solubilized in 50 mM NaOH, 1 mM dithiothreitol (DTT), 1 mM PMSF and centrifuged for 10 min at 12,850 × *g*, 4 ˚C. The soluble protoxins were recovered in the supernatant. For protoxin activation we first adjusted the pH to ~8.5 by adding ¼ volume of 1 M Tris HCl pH 8.0. Protoxins were incubated with trypsin from bovine pancreas (Sigma-Aldrich, St Louis, MO, USA) in a mass ratio of 1:20 w/w (1 h, at 37 ºC). A final concentration 1 mM of PMSF was added to stop proteolysis and proteins were dialyzed against 0.02 M Na_2HPO_4 buffer, pH 8, at 4 °C, for 16 h. Bradford assay were used to determine protein concentration (Biorad, Hercules, CA, USA), and protein integrity was analyzed through SDS-PAGE. Molecular weight markers were Precision Plus Protein Standards All Blue (Bio-Rad) and molecular masses are indicated in kDa (Fig. S1).

Bioassays. *Ae. aegypti* were reared for more than 15 years at Instituto de Biotecnología (UNAM) facilities, at 28 ºC, 75% humidity, with a 12 h: 12 h, light: dark, photoperiod. Larvae were fed commercial cat food and adults artificially fed with cow blood treated with 0.5 mg/ml heparin (Sigma) set in a Petri dish covered with a Parafilm™ membrane (Sigma-Aldrich, St Louis MO). Groups of five 3rd instar larvae in quintuplicate were placed in 2 ml of dechlorinated water using 24-well tissue culture plates (1.76 cm diameter/well) and food was not administrated during the bioassay. Larvae were treated with different toxin protein concentrations (100 to 10,000 ng/ml, determined in Bradford assays) of spore/crystal suspensions, soluble protoxins, or soluble activated toxins. A mixture of Cry11Aa and Cyt1Aa proteins (1:1 ratio) was also analyzed at the same concentrations using crystal

inclusions, protoxins or activated toxins as indicated in the text. Negative control (dechlorinated water) was included in the bioassay. Mortality was recorded after 24 h. Each bioassay was repeated three times and the medium concentration to kill 50 % of the exposed larvae (LC_{50}) was estimated by Probit analysis using Polo Plus Probit and Logit Analysis version 1.0 LeOra software. The synergism factor was determined according to Tabashnik's equation that assumes a simple additive effect [25] by dividing the theoretical toxicity by the observed toxicity of the bioassays performed with 1:1 mixtures of Cry11Aa and Cyt1Aa proteins.

Confocal microscopy. The soluble Cry11Aa protoxins and activated toxins, or purified Cry11Aa crystal inclusions were labeled with the fluorescent dye Alexa546 (Fig. S1) according to the manufacturer's instructions (Thermo Fisher Scientific, Waltham, USA). The Cyt1Aa proteins (protoxins or crystal inclusions) were labeled with the fluorescent dye Alexa647. The efficiency of labeling was measured using the molar extinction coefficient of each probe and the following equation:

$$\frac{A_x}{\varepsilon} \times \frac{\text{MW of protein}}{\text{mg protein/ml}} = \frac{\text{moles of dye}}{\text{moles of protein}} \qquad \text{(Eq. 1)}$$

where: A_x, corresponds to the maximum wavelength of dye absorbance, 554 nm for Alexa Fluor-546 and 650 nm for Alexa Fluor-647; and ε, corresponds to the molar extinction coefficient of Alexa Fluor-546 that is 203,000 M^{-1} cm^{-1} at 554 nm and for Alexa Fluor-647 is 270,000 M^{-1} cm^{-1} at 650 nm. Purity of these proteins was analyzed on SDS-PAGE with 15 % acrylamide. The labeling of the proteins was visualized by excitation of the SDS-PAGE gel with Epi-RGB light transilluminator and analyzed in an Amersham Imager 600 (GE, Irving, TX).

Second instar *Ae. aegypti* larvae were fed several days with cooked oats (25 mg/L boiled 5 min in water), to avoid auto fluorescence of the normal diet (cat food). When reached early 4th instar, larvae were placed into 24-well tissue culture plates at room temperature containing 100 ng/ml or 1000 ng/ml of labeled proteins Cry11Aa or Cyt1Aa (soluble protoxin or crystal proteins) for different exposure times (3, 6, 9 and 24 h) in the dark. In the 1:1 mixture of Cyt1Aa with Cry11Aa crystal inclusions, 100 ng/ml of each labeled crystal protein was used, while mixture of Cry11Aa-E97A with Cyt1Aa crystals was used at 1000 ng/ml and 500 ng/ml, respectively. The midgut tissue was dissected from the larvae at the indicated times and was fixed overnight in 4% paraformaldehyde supplemented with 5% sucrose in PBS pH 7.4. These midgut tissues were carefully washed three times with PBS and labeled with 4',6-diamidino-2-fenilindol (DAPI) (Sigma-Aldrich, Saint Louis, MO) at 5 mg/ml, final concentration in PBS, for 30 min. After DAPI labeling, the tissues were washed again with PBS, placed on microscopic slides and covered with Prolong Glass antifade mountant (Invitrogen, Thermo Fisher Scientific, Waltham, USA). We used two strips of masking tape in each side of the slide glass to protect the tissue to be pressed by the cover glass. Proteins labeled with Alexa546 were excited at 543 nm and fluorescence emission was analyzed at 568 nm. Alexa647-conjugated proteins were excited at 633 nm and fluorescence emission was recorded at 665 nm. Images were obtained using confocal laser scanning microscope (Olympus FV1000) at the Laboratorio Nacional de Microscopía Avanzada located in the Instituto de Biotecnología-UNAM facilities. All figures showing the whole gut are composite images constructed with Fiji-Image J software (using BigStitcher plugin) [59]. A total of five larvae were dissected for each condition and each assay was performed at least three times. The figures shown are representative images of the obtained results.

Analysis of the fluorescence intensity

The intensity of fluorescence (IF) of labeled proteins at the cytoplasm region of infected gut cells was quantified from confocal images as a proxy of protein abundance. Squares of 20 x 20 μm covering a region of interest (ROI) within the cytoplasmic region were selected from different cells (up to 14 cells per experimental treatment). Reported IF were computed with image J software [59]. Statistical analyses were performed with multiple comparison test after Kruskal-Wallis. Differences with P value < 0.001 were considered as statistically significant.

Super-Resolution radial fluctuation (SRRF) Microscopy

Sub-diffraction images were derived from the SRRF approach [26, 60]. Each super-resolution image was generated from the analysis of 100 confocal images collected with a spinning disk confocal microscope (Yokogawa CSU-W1) mounted on an epifluorescence microscope (Zeiss Observer Z.1). Detection of the signal was via a 60X S objective, numerical aperture: 1.3 (oil immersion) and an electron multiplying-CCD camera (Andor Ixon 3 EMCCD, model DU-897E-CS0-BV, Andor Technology) configured on a full chip mode. Images were collected at 2.4 sec intervals using 3i proprietary TTL synchronization electronics and SlideBook software. Fluorescence excitation was performed alternating the laser lines illumination (561, 405 and 640 nm) on a per-frame basis with 0.2, 0.4 and 0.5 sec as exposure times, respectively, and camera gain parameters (x300).

Serial stacks of the 100 images collected at the same imaging plane, were analyzed using the NanoJ-core and NanoJ-SRRF plugins of Image J [59]. The SRRF-approach encompasses two steps of image processing. First, each diffraction-limited image is

subjected to a spatial analysis, on a sub-pixel base, computing the spatial derivatives of the fluorescence field, to build a radiality map (RM) with increased resolution. The RMs were then multiplied by the corresponding fluorescence intensity to recover information about molecules abundance using the NanoJ-SRRF plugin using ring radius 0.5, radiality magnification 10, and axes in ring 8 parameters.

In the second step, the RMs were subjected to temporal analysis using NanoJ-SRRF plugin of imageJ [59]. We used the SOFI approach [61] which increases even further the resolution, through analyzing the temporal fluorescence fluctuations of the fluorophores. When computing the temporal auto-correlation signal of each pixel, the fluorescence signal contribution of the emitters to the neighboring pixels will, nonlinearly, decrease the amplitude of their auto-correlation function, hence leading to an increased resolution. By following this approach, stack of the 100 frames of RMs were integrated on a super-resolution image, computing at each pixel, the second order cumulant of the temporal auto-correlation function (TRAC2) option activated on the advanced settings of NanoJ-SRRF plugin [59].

Quantification of nanoscopic protein distributions at apical membranes

To study the relative distances of the protein distributions, ROIs of 5 μm x 10 μm were selected from their super-resolution images of apical membranes. Each ROI was manually rotated at ImageJ, using bicubic interpolation, to finally set the signal of both fluorescence channels at the horizontal, i.e. the fluorescence of Cry11Aa and Cyt1Aa proteins distributions at the apical membrane orientated along to the horizontal axis. Each ROI was exported in R statistical software using tiff library as a two channels tiff image and saved for further analysis. These images were analyzed as intensity carpets, with columns

representing nanoscopic replicates (∂r = 25 nm width) of line profiles of the fluorescence intensity distribution of each protein layer.

Stretching and folding of both protein distributions was observed at the nanoscale regimes. Hence, intensity line profiles carpets were aligned, by computing their paired correlation function:

$$pCF(y, \partial r) = \frac{<F(y,0)F(y+\partial y,\partial r)>}{<F(y,0)><F(y,\partial r)>} - 1 \qquad \text{(Eq. 2)}$$

With $F(y, \partial r)$ representing a line intensity profile of the fluorescence distribution of Cyt1Aa-Alexa647 computed at the y axis, which was defined to be perpendicular to the apical membrane (at the y axis). $F(y, 0)$ corresponds to the reference intensity profile of the same carpet. Notation $< \cdots >$ indicate the expected value, i.e.$< F >$ represents the mean value of the fluorescence intensity. The argument of the maxima of the pCF, $\arg\max(pCF)$, was then used to identify the shifts between local maxima of the line profiles of a given carpet, each separated at a ∂r distance from the reference line profile. Those shifts were used to align both Cyt1Aa-Alexa647 (F_1), and Cry11Aa-Alexa546 (F_2) florescence carpets or Cyt1Aa-Alexa647 (F_1), and Cry11Aa-E97E-Alexa546 (F_2) florescence carpets. An average representation of hundreds of line profiles aligned accordingly, including up to 10 cellular replicates of each experimental condition, is shown in Figures 7C and 7D. Confidence intervals represent standard error of the mean estimated at the level of cellular replicates.

The local distance between both Cyt1Aa and Cry11Aa protein layers was assessed from their corresponding paired cross correlation function:

$$pCCF(y, \partial r) = \frac{<F_1(y,0)F_2(y+\partial y,\partial r)>}{<F_1(y,0)><F_2(y,\partial r)>} - 1 \qquad \text{(Eq. 3)}$$

The argument of the maxima of the pCCF, $\arg\max(pCCF)$, was then used to identify distances between local maxima.

Spatial Statistics

Each data associated with a protein in a super-resolution microscopy image has a coordinate (x, y) attached to it to make a pattern of points, providing valuable information about the variables and processes collected in these points. Thus the characterization of a dot pattern inside the cells is therefore of interest for the description of some intra-cellular processes and could be carried out through spatial analysis statistics of the super-resolution microscopy images and descriptors that define the structure of the same.

The spatial analysis implemented in this work seek recognition of three types of point structures: i) concentrated, with a high density of points in specific areas of the image; ii) scattered, the points tend to occupy most of the study area maximizing the distance between points, and (iii) established randomly, there is no pattern at the points in the image.

If a group of points is distributed randomly, by example using a Poisson process with density λ, the expected number of points in a circle radius d is $\lambda \pi d^2$. Deviations from randomness can be quantified by the Ripley's K-function, which reflect the type, intensity and range of the spatial pattern by analyzing the existing distances between all points. In general terms, the Ripley's K-function can be computed as the average number of events (particles) located within a predefined radius, which is normalized for the event intensity (density) over the whole field of view (the entire image), including all potential event locations.

We made use of a modified Ripley K function proposed by Amgad *et al.* [27], which allow the identification of overlapping events at extremely high event densities, an

approach suitable to quantitate protein aggregation in fluorescent microscopic images [27]. The function used in this study can be formalized as follows:

$$K(r,n) = \frac{|\Omega|}{n(n-1)} \sum_{i=1}^{N_p} \frac{\pi r^2}{A_{xr}} P_x \left(P_x - 1 + \sum_{x \neq y}^{N_p} I_r(dxy) \right) \quad \text{(Eq. 4)}$$

As our objects of study (protein distributions) are represented as intensity values on a super-resolution microscopy image, the intensity unit can be considered as a proxy of the number particles, i.e. in an 8-bit gray scale image, the saturation limit is taken as $2^8 - 1 = 255$ particles per pixel. Hence, the number of particles around any particle within the same pixel is $P_x - 1$, with P_x being the intensity at pixel x. N_p is defined as the total number of pixels and n is the total fluorescence intensity over the whole field of view. n is also indicative of the total number of events within the study area and λ is the intensity (density) of events. Such that $\lambda = \frac{n}{|\Omega|}$, and $|\Omega|$ is the area of the study region (the entire field of view, including all potential event locations). A_{xr} is defined as the area of the portion of circle $b(x,r)$, centered on position x and having a searching radius r. The later defines the boundary radius of the region under study. The distance between event locations x and y is defined as dxy, and I_r is an indicator function that takes a value of 1 if dxy is less or equal to r (the searching radius), or a value of 0 otherwise.

It is important to mention that edge effects can be particularly challenging when it comes to Ripley's K-function calculation. The $K(n,r)$ metrics proposed by Amgad *et al.* [27], also encompass the Besag´s correction [62], one of the best and most widely accepted methods to correct for this kind of bias. That is, the overall number of events within a particular r is divided by the proportion of the area of a circle of the same radius (centered on the central event) that is included within the field of view. The Besag's correction term

is given by $A_{xy} = b(x,r) \cap |\Omega|$. Not that at the edges, the area $b(x,r)$,is less than πr^2, due to pixilation effects.

Finally, to simply testing about complete randomness we further used an implementation of Ripley's K-function that normalizes for the event density and have unit variance. As proposed by Lagache et al [63], it has the following equation:

$$\widetilde{K}(r,n) = \frac{K(r,n) - \pi r^2}{\sqrt{var\{K(k,n)\}}} \quad \text{(Eq. 5)}$$

Once the $\widetilde{K}$ function is computed, it is compared to its upper and lower boundary limits, (typically, the 1st and 99th quantiles), as proposed by Lagache et al [63] to assess of what its value would be in a complete spatial randomness, or not.

Interpretations about the meaning of the $\widetilde{K}$ metrics can be as follows: when the observed $\widetilde{K}$ value is greater than the expected $\widetilde{K}$ value for randomness, at a particular r distance, the distribution is considered as clustered at that distance. When the observed $\widetilde{K}$ value is less than the expected $\widetilde{K}$ value for randomness, the distribution is dispersed at that distance. When the observed $\widetilde{K}$ value is greater than the confidence interval, the spatial clustering at that distance is statistically significant. When the observed $\widetilde{K}$ value is lower than the confidence interval, the spatial dispersion at that distance is statistically significant.

Calculations of $\widetilde{K}$ were made over the super-resolution images using a MATLAB script provided by Amgad *et al.* [27] (MATLAB R2020b). At least 14 squares of 10 x 10 µm from the cytoplasm of the super-resolution images of different cells of each condition were analyzed. ROIs super-resolution images were separated in single channels, exported in TIF format (8-bits, gray scale images) and used as input element for the MATLAB script. The $\widetilde{K}$ metric was computed for each fluorescence channel separately at different

radii r at steps of $\Delta r = 25$ nm, with their respective critical quantiles (Figs. 6D, 6E, 6G, 7F and 7G). Statistical analyses were performed over the $\arg\max(\widetilde{K})$ considering multiple comparison test after Kruskal-Wallis (Figs. 6F, 7F and 7G). A P value < 0.05 was considered as statistically significant.

ACKNOWLEDGEMENTS

This work was supported by Dirección General de Asuntos del Personal Académico de la Universidad Nacional Autónoma de México-DGAPA/UNAM IN202718 and DGAPA/UNAM IN203619, National Institutes of Health-NIH 2R01 AI066014.

The authors thank the teams from insectarium of IBT-UNAM (Lizbeth Cabrera) and LNMA-UNAM personal (Andres Saralegui, Xochitl Alvarado and Arturo Pimentel) for their technical support.

References

1. Pardo-López L, Soberón M, Bravo A. *Bacillus thuringiensis* insecticidal 3-domain Cry toxins: mode of action, insect resistance and consequences for crop protection. FEMS Microbiology Reviews. 2013; 37: 3-22.

2. Bravo A, Likitvivatanavong S, Gill SS, Soberón M. *Bacillus thuringiensis*: a story of a successful bioinsecticide. Insect Biochemistry and Molecular Biology. 2011; 41: 423-431.

3. Ben-Dov E. *Bacillus thuringiensis* subs. *israelensis* and its dipteran-specific toxins. Toxins. 2014; 6: 1222-1243.

4. Berry C, O'Neil S, Ben-Dov E, Jones AF, Murphy L, Quail MA, Holden MT, Harris D, Zaritsky A, Parkhill J. Complete sequence and organization of pBtoxis, the toxin-coding plasmid of *Bacillus thuringiensis* subsp. *israelensis*. Applied and Environmental Microbiology. 2002; 68: 5082-5095.

5. Soberón M, Lopez-Diaz JA, Bravo A. Cyt toxins produced by *Bacillus thuringiensis:* a protein fold conserved in several pathogenic microorganisms. Peptides. 2013; 41: 87-93.

6. Li J, Koni PA, Ellar DJ. Structure of the mosquitocidal δ-endotoxin CytB from *Bacillus thuringiensis* sp. *kyushuensis* and implications for membrane pore formation. Journal Molecular Biology. 1996; 257: 129-152.

7. Cohen S, Albeck S, Ben-Dov E, Cahan R, Firer M, Zaritsky A, Dym O. Cyt1Aa toxin: crystal structure reveals implications for its membrane-perforating function. Journal Molecular Biology. 2011; 413: 804-814.

8. Soberón M, Fernández LE, Pérez C, Gill SS, Bravo A. Mode of action of mosquitocidal *Bacillus thuringiensis* toxins. Toxicon. 2007; 49: 597-600.

9. Wirth MC, Park HW, Walton WE, Federici BA. Cyt1A of *Bacillus thuringiensis* delays evolution of resistance to Cry11A in the mosquito *Culex quinquefasciatus*. Applied and Environmental Microbiology. 2005; 71: 185-189.

10. Wirth MC, Georghiou GP, Federici BA. CytA enables CryIV endotoxins of *Bacillus thuringiensis* to overcome high levels of CryIV resistance in the mosquito, *Culex quinquefasciatus.* Proceedings of the National Academy of Sciences USA. 1997; 94: 10536-10540.

11. Wirth MC, Federici BA, Walton WE. Cyt1A from *Bacillus thuringiensis* synergizes activity of *Bacillus sphaericus* against *Aedes aegypti* (Diptera: Culicidae). Applied and Environmental Microbiology. 2000; 66: 1093-1097.

12. Pérez C, Fernandez LE, Sun J, Folch JL, Gill SS, Soberón M, Bravo A. *Bacillus thuringiensis* subsp. *israelensis* Cyt1Aa synergizes Cry11Aa toxin by functioning as a membrane-bound receptor. Proceedings of the National Academy of Sciences USA. 2005; 102: 18303-18308.

13. Pérez C, Muñoz-Garay C, Portugal LC, Sánchez J, Gill SS, Soberón M, Bravo A. *Bacillus thuringiensis* ssp. *israelensis* Cyt1Aa enhances activity of Cry11Aa toxin by facilitating the formation of a pre-pore oligomeric structure. Cellular Microbiology. 2007; 9: 2931-2917.

14. Cantón PE, Zanicthe Reyes EZ, Ruiz de Escudero I, Bravo A, Soberón M. Binding of *Bacillus thuringiensis* subsp. *israelensis* Cry4Ba to Cyt1Aa has an important role in synergism. Peptides. 2011; 32: 595-600.

15. Fernández-Luna MT, Tabashnik B., Lanz-Mendoza H., Bravo A., Soberón M., Miranda-Rios J. Single-concentration tests show synergism among *Bacillus thuringiensis* subsp. *israelensis* toxins against the malaria vector mosquito *Anopheles albimanus*. Journal Invertebrate Pathology. 2010; 104: 231-233.

16. Ravoahangimalala O, Charles J-F. In vitro binding of *Bacillus thuringiensis* var. *israelensis* individual toxins to midgut cells of *Anopheles gambiae* larvae (Diptera:Culicidae). FEBS Letters. 1995; 362: 111-115.

17. Fernández LE, Aimanova KG, Gill SS, Bravo A, Soberón M. A GPI-anchored alkaline phosphatase is a functional midgut receptor of Cry11Aa toxin in *Aedes aegypti* larvae. Biochemical Journal. 2006; 394: 77-84.

18. Chen J, Aimanova K, Gill SS. Functional analysis of *Aedes aegypti* alkaline phosphatase ALP1 involved in the toxicity of Cry toxins from *Bacillus thuringiensis* subsp. *israelensis* and *jegathesan* Peptides. 2017; 98: 78-85.

19. Chen J, Likitvivatanavong S, Aimanova KG, Gill SS. A 104 kDa *Aedes aegypti* aminopeptidase N is a putative receptor for the Cry11Aa toxin from *Bacillus thuringiensis* subs. *israelensis*. Insect Biochemistry and Molecular Biology. 2013; 43: 1201-1208.

20. Chen J, Aimanova KG, Gill SS. *Aedes aegypti* cadherin receptor that mediates *Bacillus thuringiensis* Cry11Aa toxicity is essential for mosquito development. PLoS Neglected Tropical Diseases 2020; 14: e0007948.

21. Schnell DJ, Pfannenstiel MA, Nickerson KW. Bioassays of solubilized *Bacillus thuringiensis* var. *israelensis* crystals by attachment to latex beads. Science. 1984; 223: 1191-1193.

22. Torres-Quintero M, Gómez I, Pacheco S, Sánchez J, Flores H, Osuna J, Mendoza G, Soberón M, Bravo A. Engineering *Bacillus thuringiensis* Cyt1Aa toxin specificity from dipteran to lepidopteran toxicity. Scientific Reports. 2018; 8: 4989.

23. Muñóz-Garay C, Rodríguez-Almazán CR, Aguilar JN, Portugal L, Gómez I, Saab-Rincon G, Soberón M, Bravo A. Oligomerization of Cry11Aa from *Bacillus thuringiensis* has an important role in toxicity against *Aedes aegypti*. Applied and Environmental Microbiology. 2009; 75: 7548-7550.

24. López-Diaz JA, Cantón PE, Gill SS, Soberón M, Bravo A. Oligomerization is a key step in Cyt1Aa membrane insertion and toxicity but not necessary to synergize Cry11Aa toxicity in *Aedes aegypti* larvae. Environmental Microbiology. 2013; 15: 3030-3039.

25. Tabashnik BE. Evaluation of synergism among *Bacillus thuringiensis* toxins. Applied and Environmental Microbiology. 1992; 58: 3343–3346.

26. Culley S, Tosheva KS, MatosPereira P, Henriques R. SRRF: Universal live-cell super-resolution microscopy. The International Journal of Biochemistry and Cell Biology. 2018; 101: 74-79

27. Amgad M, Itoh A, Tsui MMK. Extending Ripley's K-function to quantify Aggregation in 2-D grayscale images. Plos ONE. 2015; 10: e0144404.

28. Behemer ST. Insect herbivore nutrient regulation. Annual Review of Entomology. 2009; 54: 165-187.

29. Gore J, Adamczyk JJ, Blanco C. Selective feeding of tobacco budworm and bollworm (Lepidoptera: Noctuidae) on meride diet with different concentrations of *Bacillus thuringiensis* proteins. Journal of Economic Entomology. 2005; 95: 763-769.

30. Singh G, Rup PJ, Koul O. Selective feeding of *Helicoverpa armigera* (Hübner) and *Spodoptera litura* (Fabricius) on meride diet with *Bacillus thuringiensis* toxins. Journal of Insect Behavior. 2008; 21: 407-421.

31. Orpet RJ, Degain BA, Tabashnik BE, Carrière Y. Balancing Bt toxin avoidance and nutrient intake by *Helicoverpa zea* (Lepidoptera: Noctuidae). Journal of Economic Entomology. 2015; 108: 2581-2588.

32. Walker ED. Effect of low temperature on feeding rate of *Aedes stimulans* larvae and efficacy of *Bacillus thuringiensis* var. *israelensis* (H-14). Journal of the American Mosquito Control Association. 1995; 11: 107–110.

33. Jiménez-Juárez N, Muñoz-Garay C, Gómez I, Saab-Rincon G, Damian-Alamazo JY, Gill SS, Soberón M, Bravo A. *Bacillus thuringiensis* Cry1Ab mutants affecting

oligomer formation are non toxic to *Manduca sexta* larvae. Journal of Biological Chemistry 2007; 282: 21222-21229.

34. Nascimento NA, Torres-Quintero MC, López-Molina S, Pacheco S, Romão TP, Pereira-Neves A, Soberón M, Bravo A, Silva-Filha MHNL. Functional *Bacillus thuringiensis* Cyt1Aa is necessary to synergize *Lysinibacillus sphaericus* Binary toxin against Bin-resistant and refractory mosquito species. Applied and Environmental Microbiology. 2020; 86: e02770-19

35. Krishnamoorthy M, Jurat-Fuentes JL, McNall RJ, Andacht T, Adang MJ. Identification of novel Cry1Ac binding proteins in midgut membranes from *Heliothis virescens* using proteomic analyses. Insect Biochemistry and Molecular Biology. 2007; 37: 189-201.

36. Chen L-Z, Liang G-M, Zhang J, Wu K-M, Guo Y-Y, Rector BG. Proteomic analysis of novel Cry1Ac binding proteins in *Helicoverpa armigera* (Hübner). Archives of Insect Biochemistry and Physiology. 2010; 73: 61-73.

37. Bayyareddy K, Andacht TM, Abdullah MA, Adang MJ. Proteomic identification of *Bacillus thuringiensis* subsp *israelensis* toxin Cry4Ba binding proteins in midgut membranes from *Aedes* (*Stegomyia*) *aegypti* Linnaeus (Diptera, Culicidae) larvae. Insect Biochemistry and Molecular Biology. 2009; 39: 279–286.

38. Cancino-Rodezno A, Lozano L, Oppert C, Castro JI, Lanz-Mendoza H, Encarnación S, Evans AE, Gill SS, Soberón M, Jurat-Fuentes JL, and Bravo A. Comparative proteomic analysis of *Aedes aegypti* larval midgut after intoxication with Cry11Aa toxin from *Bacillus thuringiensis*. PloS ONE. 2012; 7: e37034

39. Los FCO, Kao Ch-Y, Smitham J, McDonald KL, Ha Ch, Peixoto ChA, Aroian RV. RAB-5 and RAB-11-dependent vesicle-trafficking pathways are required for plasma

membrane repair after attack by bacterial pore-forming toxin. Cell Host Microbe. 2011; 9: 147–157.

40. Peraro M, van der Goot F. Pore-forming toxins: ancient, but never really out of fashion. Nature Reviews in Microbiology 2016; 14: 77–92.

41. Bischofberger M, Iacovache I, van der Goot FG. Pathogenic pore-forming proteins: function and host response. Cell Host Microbe. 2012; 12: 266–275.

42. Rouis S, Chakroun M, Saadaoui I, Jaoua S. Proteolysis, histopathological effects, and immunohistopathological localization of δ-Endotoxins of *Bacillus thuringiensis* subsp. *kurstaki* in the midgut of lepidopteran olive tree pathogenic insect *Prays oleae*. Molecular Biotechnology. 2007; 35: 141-148.

43. BenFarhat-Touzri D, Saadaoui M, Abdelkefi-Mesrati L, Saadaoui I, Azzouz H, Tounsi S. Histopathological effects and determination of the putative receptor of *Bacillus thuringiensis* Cry1Da toxin in *Spodoptera littoralis* midgut. Journal of Invertebrate Pathology. 2013; 112: 142–145.

44. Delello E, Hanton WK, Bishoff ST, Misch DW. Histopathological effects of *Bacillus thuringiensis* on the midgut of tobacco hornworm larvae (*Manduca sexta*): Low doses compared with fasting. Journal of Invertebrate Pathology. 1984; 43: 169-181.

45. Uzunhisarcikli M, Kalender Y, Suludere Z. Effects of *Bacillus thuringiensis kurstaki* on Midgut Cells of *Thaumetopoea pityocampa* (Lepidoptera: Thaumetopoeidae), The Pine Processionary Caterpillar. Gazi University Journal of Sciences. 2014; 27: 861-870.

46. Castro BMCE, Martinez LC, Barbosa SG, Serrão JE, Wilcken CF, Soares MA, da Silva AA, de Carvalho AG, Zanuncio JC. Toxicity and cytopathology mediated by *Bacillus thuringiensis* in the midgut of *Anticarsia gemmatalis* (Lepidoptera: Noctuidae). Scientific Reports. 2019; 9: 6667

47. Bravo A, Jansens S, Peferoen M. Immunocytochemical localization of *Bacillus thuringiensis* insecticidal crystal proteins in intoxicated insects. Journal of Invertebrate Pathology. 1992; 60: 237-247.

48. Bauer LS, Pankratz S. Ultrastructural Effects of *Bacillus thuringiensis* var. San Diego on midgut cells of the cottonwood leaf beetle. Journal of Invertebrate Pathology. 1992; 60: 15-25.

49. Ahmed AM, Hussein HI, El-Kersh TA, Al-Sheikh YA, Ayaad TH, El-Sadawy HA, Al-Mekhlafi F., Ibrahim MS, Al-Tamimi J, Nasr FA. Larvicidal activities of indigenous *Bacillus thuringiensis* isolates and nematode symbiotic bacterial toxins against the mosquito vector, *Culex pipiens* (Diptera: Culicidae). Journal of Arthropod-Borne Diseases. 2017; 11: 260–277.

50. Ruiz LM, Segura C, Trujillo J, Ordúz S. In vivo binding of the Cry11Bb toxin of *Bacillus thuringiensis* subsp. *medellin* to the midgut of mosquito larvae (Diptera: Culicidae). Memórias do Instituto Oswaldo Cruz. 2004; 99: 73-79.

51. Lavarías S, Arrighetti F, Siri A. Histopathological effects of cypermethrin and *Bacillus thuringiensis* var. *israelensis* on midgut of *Chironomus calligraphus* larvae (Diptera: Chironomidae). Pesticide Biochemistry and Physiology. 2017; 139: 9–16.

52. Cavados CFG, Majerowicz S, Chaves JQ, Araújo-Coutinho CJPC, Rabinovitch L. Histopathological and ultrastructural effects of δ-endotoxins of *Bacillus thuringiensis* serovar *israelensis* in the midgut of *Simulium pertinax* larvae (Diptera, Simuliidae). Memórias do Instituto Oswaldo Cruz. 2004; 99: 493-498.

53. Zhuang M, Oltean DI, Gómez I, Pullikuth AK, Soberón M, Bravo A, Gill S.S. *Heliothis virescens* and *Manduca sexta* lipid rafts are involved in Cry1A toxin binding to the

midgut epithelium and subsequent pore formation. Journal Biological Chemistry. 2002; 277: 13863-13872.

54. Wu D, Johnson JJ, Federici BA. Synergism of mosquitocidal toxicity between CytA and CryIV proteins using inclusions produced from cloned genes of *Bacillus thuringiensis* subsp. *israelensis*. Molecular Microbiology. 1994; 13: 965-972.

55. Chang C, Yu YM, Dai SM, Law SK, Gill SS. High-level *cryIVD* and *cytA* gene expression in *Bacillus thuringiensis* does not require the 20-kilodalton protein, and the coexpressed gene products are synergistic in their toxicity to mosquitoes. Applied and Environmental. Microbiology. 1993; 59: 815–821.

56. Schaeffer P, Millet J, Aubert J-P. Catabolic repression of bacterial sporulation. Proceedings of the National Academy of Sciences USA. 1965; 54: 704-711.

57. Thomas WE, Ellar DJ. *Bacillus thuringiensis* var *israelensis* crystal delta-endotoxin: effects on insect and mammalian cells *in vitro* and *in vivo*. Journal of Cell Sciences. 1983; 60: 181–197.

58. Güereca L, Bravo A, Quintero R. Design of an aqueous two-phase system for the purification of ICP from *Bacillus thuringiensis*. Process Biochemistry. 1994; 29: 181-185.

59. Schindelin J, Arganda-Carreras I, Frise E, Kaynig V, Longair M, Pietzsch T, Preibisch S, Rueden C, Saalfeld S, Schmid B, Tinevez J-Y, White DJ, Hartenstein V, Eliceiri K, Tomancak P, Cardona A. Fiji: an open source platform for biological-image analysis. Nature Methods. 2012; 9: 676-682.

60. Gustafsson N, Culley S, Ashdown G, Owen DM, Pereira PM, Henriques R. Fast live-cell conventional fluorophore nanoscopy with ImageJ through super-resolution radial fluctuations. Nature Communications. 2016; 7: 1-9.

61. Dertinger T, Colyer R, Lyer G, Weiss S, Enderlein J. Fast, background-free, 3D super-resolution optical fluctuation imaging (SOFI). Proceedings of the National Academy of Sciences USA. 2009; 106:22287-22292.

62. Besag JE. Comments on Ripley’s paper. Journal of the Royal Statistical Society, Series B. 1977; 39: 193–195.

63. Lagache T, Lang G, Sauvonnet N, Olivo-Marin J-Ch. Analysis of the spatial organization of molecules with robust statistics. PlosONE 2013; 8: e80914.

Figure legends

Figure 1. Insecticidal activity of soluble activated toxin, soluble protoxin and crystal inclusions from Cry11Aa and Cyt1Aa, wild type or mutant proteins against 3rd instar *Aedes aegypti* larvae after 24 h of exposure. Each bioassay was repeated three times with five replicates. The LC_{50} values and confident intervals (CI) of the samples to larvae were calculated by using Polo Plus Probit and Logit Analysis version 1.0 LeOra software, as follows: Cry11Aa crystals= 329.3 (CI: 203.1-605.6) ng/ml; Cyt1Aa crystals= 679.23 (CI: 554.9-851.1) ng/ml; Cry11Aa- Cyt1Aa 1:1 crystals mixture = 51.53 (CI: 554.9-851) ng/ml; soluble Cry11Aa -Cyt1Aa 1:1 protoxins mixture= 5335 (CI: 4503-6339) ng/ml. LC_{50} could not be calculated for those samples without larvicidal activity.

Figure 2. Dissected midguts from Cry11Aa and/or Cyt1Aa-treated *Aedes aegypti* 4th instar larvae recorded by clear field microscopy. Blue arrowheads point to severely affected

gastric *caeca* region of some midguts. Representative composite images were constructed with Fiji-Image J software.

Figure 3. *In vivo* localization of labeled Cry11Aa-Alexa546 and Cyt1Aa-Alexa647, or mutant Cry11AaE97A-Alexa546 and Cyt1AaV122E-Alexa647 proteins, inside the midgut tissue of *Aedes aegypti* larvae. Individuals were fed with of each labeled protein (100 ng/ml or 1000 ng/ml) administrated as soluble activated toxin, soluble protoxin or crystal inclusions for 24 h. Midgut tissue was dissected and processed as indicated in materials and methods. Nucleus was stained with DAPI and labeled proteins were observed with confocal laser Olympus FV1000 scanning microscope. Inserts show selected images to improve clarity of the images. White arrowhead points to Cry11Aa-E97A activated toxin protein bound to microvilli membrane from medium tissue region. Orange arrowhead points to Cry11Aa protoxin internalized into small vesicles. Yellow arrowheads point to cells of the medium midgut highly brillinat due to intensive Cry11Aa internalization. Red arrowheads point to cells that were already broken down.

Figure 4. *In vivo* localization of labeled crystal inclusions from Cry11Aa-Alexa546 wild type (Panel A) or Cry11AaE97A-Alexa546 mutant (Panel B) in the midgut tissue of *Aedes aegypti* larvae. Individuals were fed (100 ng/ml or 1000 ng/ml) of each labeled crystal inclusions for 3, 6 and 9 h. Midgut tissue was dissected and processed as indicated in materials and methods. Nucleus was stained with DAPI and labeled proteins were observed with confocal laser Olympus FV1000 scanning microscope. Clear field microscopy images of *caeca* region are also shown in panels A and B of this figure. Yellow arrowheads point to highly brilliant midgut cells due to Cry11Aa-

Alexa546 internalization, that were named Cry11Aa-P2 cells. Red arrowheads point to cells that were already broken down and the blue arrow points to the gastric *caeca* region that was severely affected. Panel C shows the complete midgut tissue and a fragment from the medium midgut region showing the highly brilliant cells containing Cry11Aa-Alexa546 protein (Cry11Aa-P2 cells) and dark cells (Cry11Aa-P1). Yellow arrowheads point to Cry11Aa-P2 cells. Panel C also shows the intensity of Fluorescence (IF) measurements of 20 x 20 □m squares selected from the cytoplasm of different cells from all conditions. Data are shown as arbitrary fluorescent units (a.f.u.). Different asterisks indicate statistically significant different data analyzed by using Kruskal-Wallis test (*P* value < 0.001).

Figure 5. *In vivo* co-localization of labeled Cry11Aa and Cyt1Aa proteins during their synergism in the midgut tissue of *Aedes aegypti* larvae fed with different mixtures of the labeled crystal inclusions. Panel A shows mixtures of 1:1 [100 ng/ml] Cry11Aa-Alexa546 with Cyt1Aa-Alexa647. Panel B shows Cry11AaE97A-Alexa546 mutant [1000 ng/ml] with Cyt1Aa-Alexa647 [500 ng/ml]. Larvae were fed with labeled crystal inclusions proteins for 3 h. Next, larvae were processed, nucleus was stained with DAPI and labeled proteins were observed with confocal laser Olympus FV1000 scanning microscope. Inserts show selected images to improve clarity of the images and yellow arrowheads point to highly brilliant midgut cells due to Cry11Aa internalization Panel C shows the intensity of Fluorescence (IF) measurements of 20 x 20 μm squares selected from the cytoplasm of different cells. Data are shown as arbitrary fluorescent units (a.f.u.). Different asterisks indicate statistically significant different data analyzed by using Kruskal-Wallis test (*P* value < 0.001).

Figure 6. High resolution SRRF analysis showing the *in vivo* nanoscale localization of the labeled Cry11Aa-Alexa546 (Panel A), mutant Cry11AaE97A-Alexa546 (Panel B) and Cyt1Aa-Alexa647 (Panel C), in the midgut tissue of *Aedes aegypti* larvae. Individuals were fed with 1000 ng/ml of each labeled crystal inclusions proteins for 6 h. Next, larvae were processed, nucleus were stained with DAPI and 100 frames of raw spinning disk data were recorded with the confocal Yokogawa spinning disk microscope alternating the laser lines illumination (405, 561 and 640 nm) per-frame basis, and Images were analyzed using NanoJ-core and NanoJ-SRRF plugins of Fiji-Image J software as described in materials and methods. Panel A, shows one brilliant and one dark cell after Cry11Aa-Alexa546 ingestion. Inserts show selected images to improve clarity of the images. Panel D, shows the Ripley's K-function statistical image analysis of the *xy* pattern organization observed in selected 10 x 10 μm squares selected form different cells of larvae intoxicated with Cry11Aa-Alexa546. Insert, shows an enlargement of the distal organization. The brilliant cells are named Cry11Aa-P2, while dark cells are named Cry11Aa-P1. Similar analyses were performed for different cells of larvae intoxicated with Cry11AaE97A-Alexa546 (Panel E) or Cyt1Aa-Alexa647 (Panel G). These Ripley's K-function statistical image analysis compute the average number of particles located within a predefined radius of any typical event, normalized for the event intensity (density). Panel F, shows the Kruskal-Wallis test performed to the distributions with Cry11Aa-Alexa546 or Cry11AaE97A-Alexa546. Different asterisks indicate significant different data (*P* value= 0.05).

Figure 7. Nanoscopic *in vivo* organization of Cry11Aa or Cyt1Aa proteins during their synergism in the midgut tissue of *Aedes aegypti* larvae. Images were analyzed by high resolution SRRF microscopy. Larvae were fed 3 h with mixtures of crystals Cry11Aa-Alexa546 with Cyt1Aa-Alexa647 at 100 ng/mL of each protein (Panel A), and mutant Cry11AaE97A-Alexa546 with Cyt1Aa-Alexa647 at 1000 ng/mL + 500 ng/mL, respectively (Panel B). Larvae were processed as explained in figure 6. Panel A, shows one complete cell and one cell that is already broken down. Inserts show selected images to improve clarity of the images. Red arrowhead points to the specific Cyt1Aa-Alexa647 and Cry11Aa-Alexa546 proteins organization in apical membrane, where Cry11Aa-Alexa546 was located down facing the cell cytoplasm and Cyt1Aa-Alexa647 was found in the upper layer. Yellow arrowhead points to the inverted organization presented by Cry11AaE97A-Alexa546 mutant and Cyt1Aa-Alexa647. Panels C and D, show the quantitative line pattern analysis, where Cyt1Aa protein was arbitrary located at zero value, as described in materials and methods, from 24 squares of 2.5 x 5 μm selected from Cry11Aa-Alexa546 with Cyt1Aa-Alexa647 mixture (Panel C), or 34 squares of 2.5 x 5 μm selected from Cry11AaE97A-Alexa546 with Cyt1Aa-Alexa647 mixture (Panel D). Panel E, shows the distribution of the maximal distances of Cry11Aa and Cry11AaE97A to Cyt1Aa, which was arbitrarily located at zero value. Panels F and G, show the Ripley's K-function statistical image analysis of the *xy* pattern organization observed in 14 selected 10 x 10 μm squares selected form different cells of the larvae intoxicated with the different mixtures of proteins. Panel F, shows the analysis of the fluorescence of Cry11Aa-Alexa546 or mutant Cry11AaE97A-Alexa546 in these mixtures and Panel G, shows the analysis of the fluorescence of Cyt1Aa-

Alexa647 in these mixtures. Panels F and G, also show the Kruskal-Wallis test analyses of these distributions, different asterisks indicate significant different data (*P* value= 0.05), no asterisk indicate no statistical difference.

Figure 1

Click here to access/download;Figure;figure 1.tif

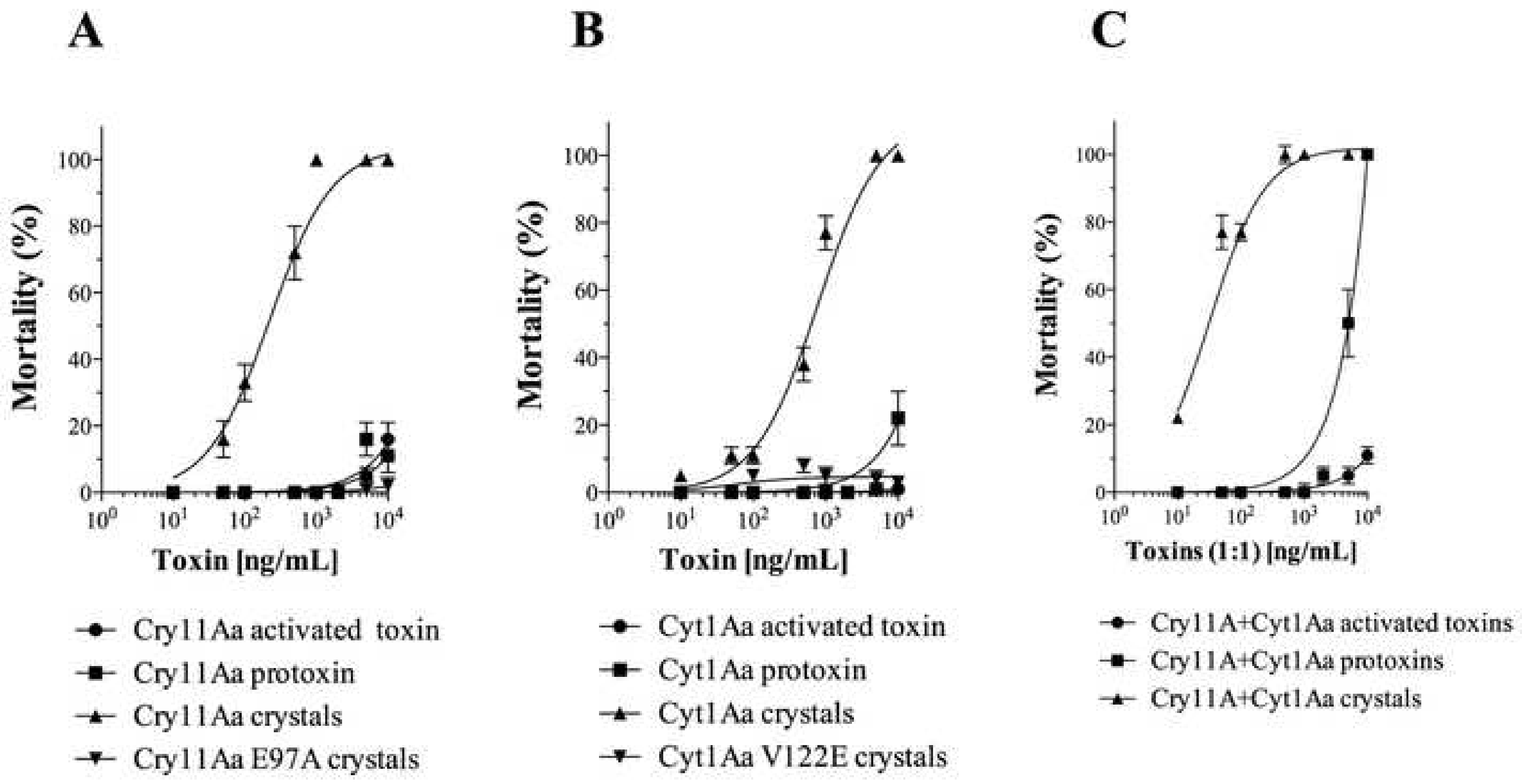

Figure 2

Click here to access/download;Figure;Figure 2.tif

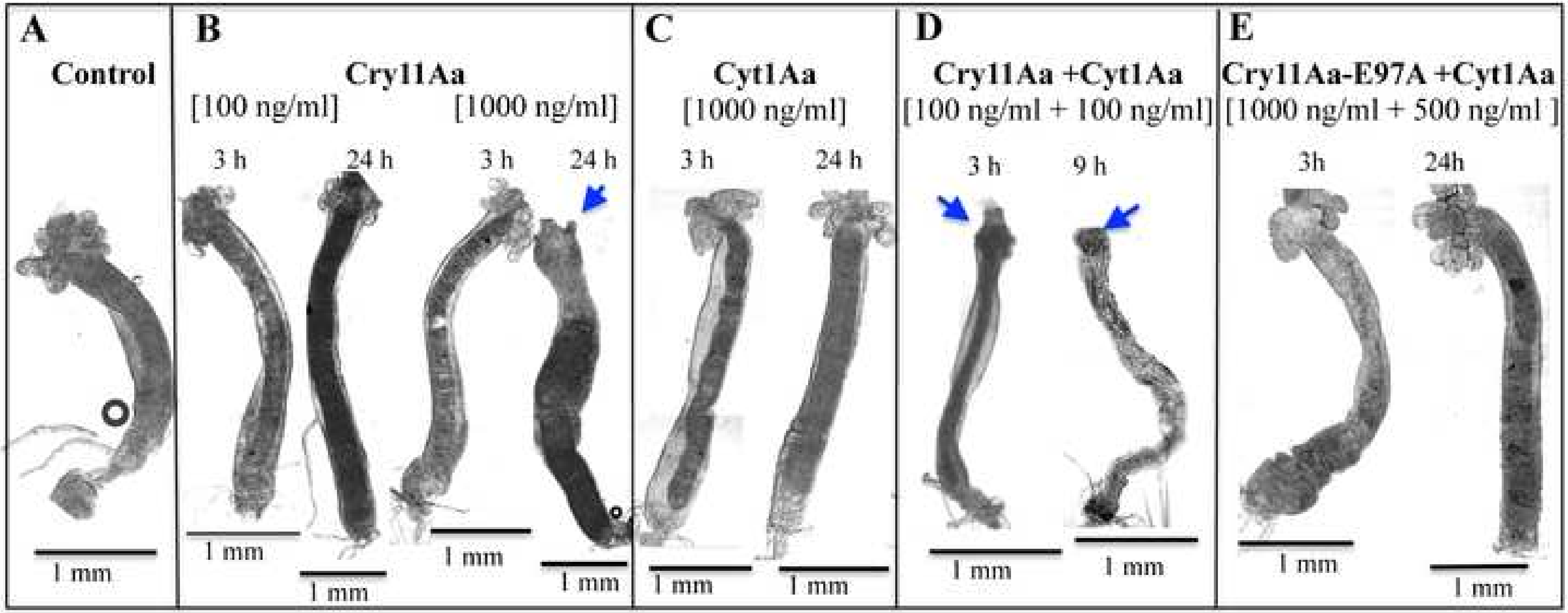

Click here to access/download;Figure;Figure 3.tif

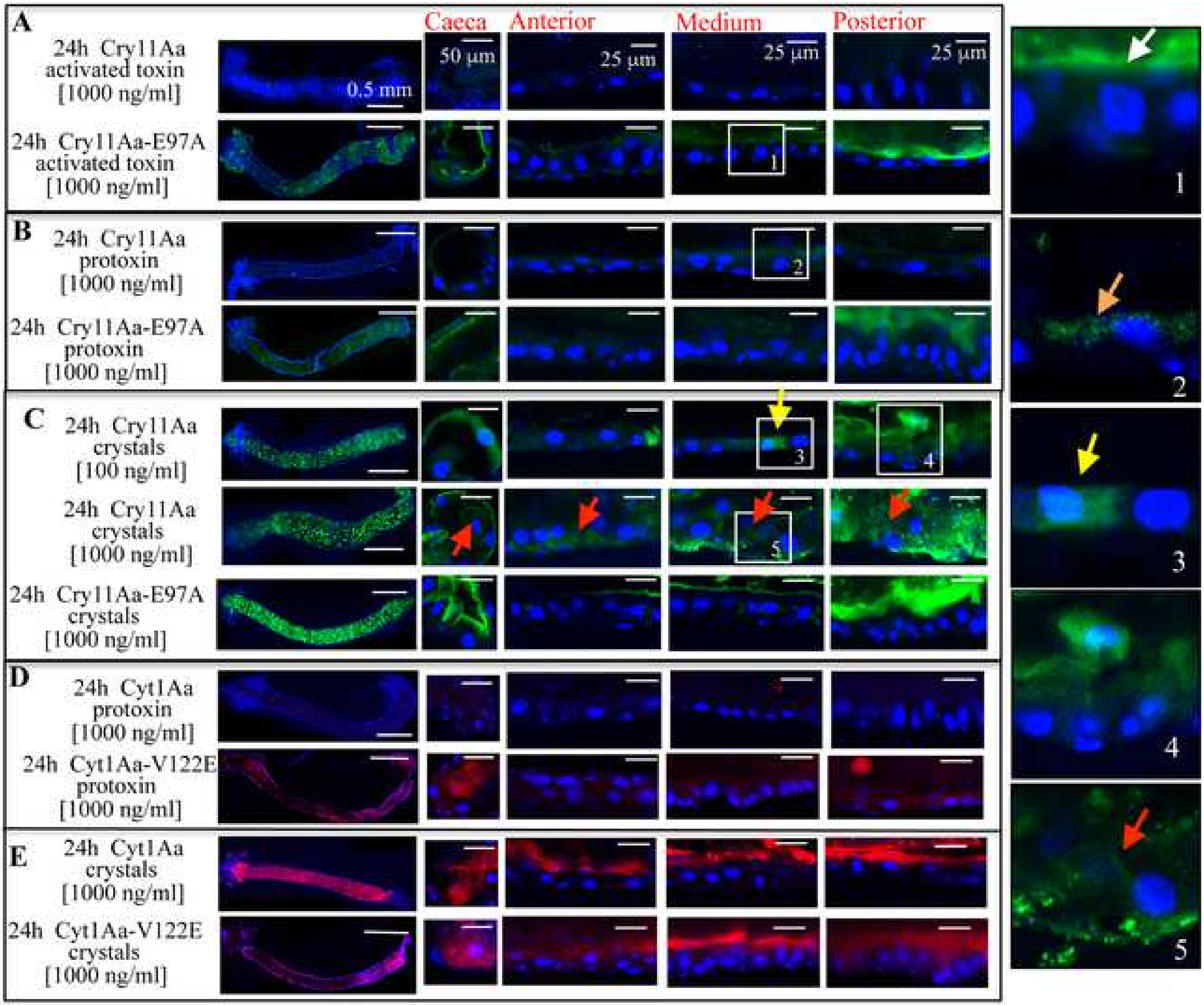

Click here to access/download;Figure;Figure 4.tif

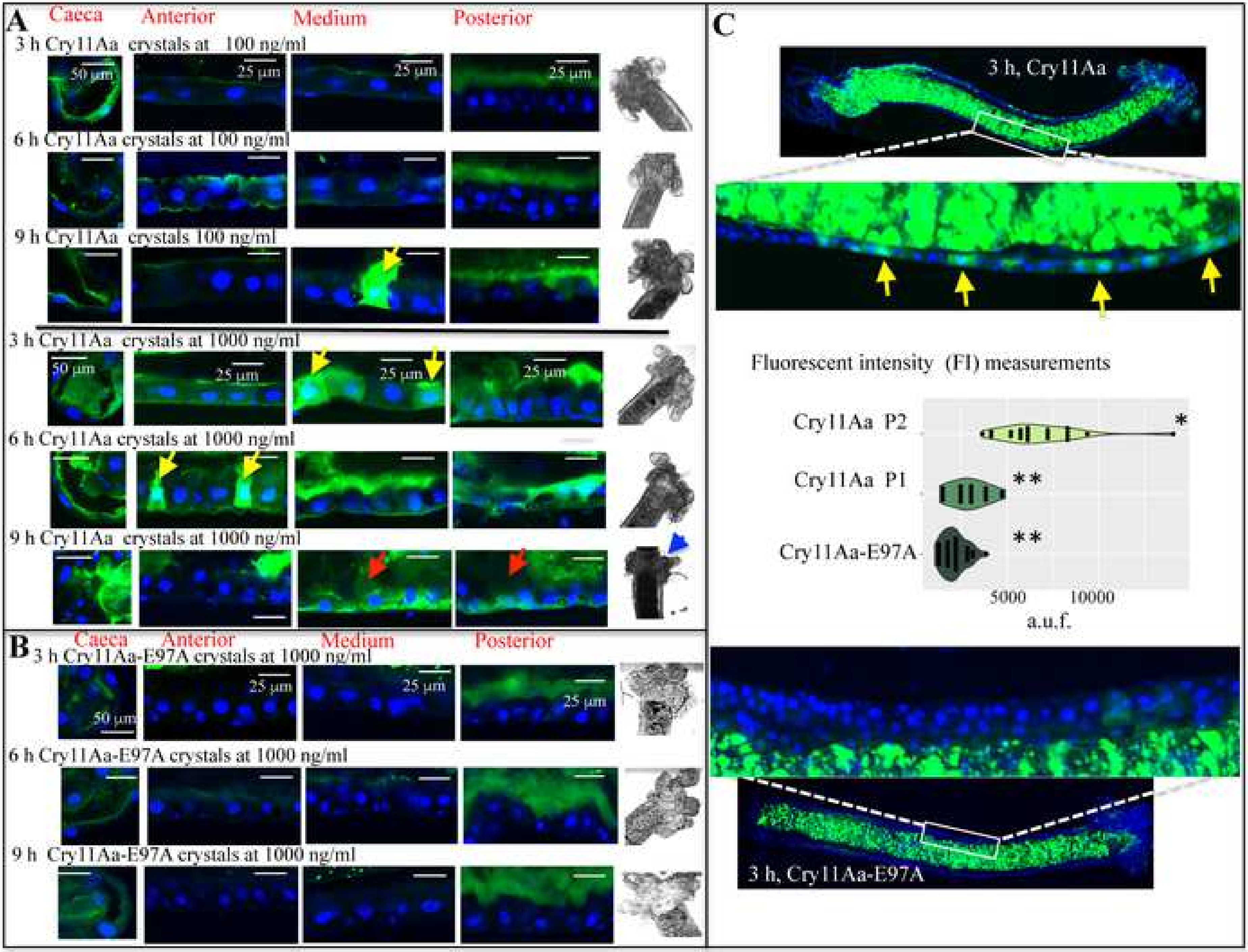

Figure 5

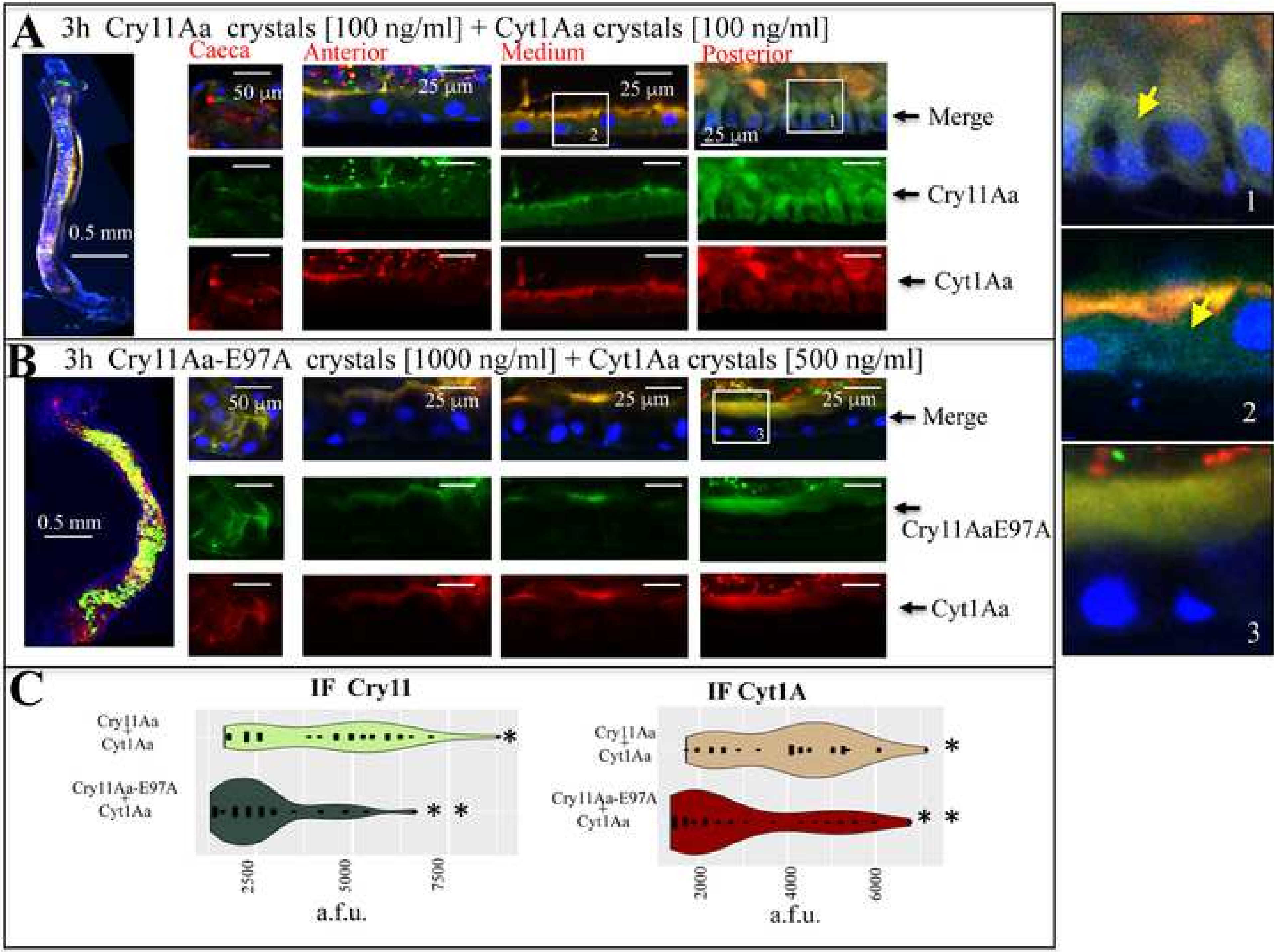

A 3h Cry11Aa crystals [100 ng/ml] + Cyt1Aa crystals [100 ng/ml]
Caeca
Anterior
Medium
Posterior
0.5 mm
50 µm
25 µm
25 µm
25 µm
Merge
Cry11Aa
Cyt1Aa
B 3h Cry11Aa-E97A crystals [1000 ng/ml] + Cyt1Aa crystals [500 ng/ml]
0.5 mm
50 µm
25 µm
25 µm
25 µm
Merge
Cry11AaE97A
Cyt1Aa
1
2
3
C
IF Cry11
Cry11Aa + Cyt1Aa
Cry11Aa-E97A + Cyt1Aa
2500
5000
7500
a.f.u.
IF Cyt1A
Cry11Aa + Cyt1Aa
Cry11Aa-E97A + Cyt1Aa
2000
4000
6000
a.f.u.

Figure 6

Click here to access/download;Figure;Figure 6.tif

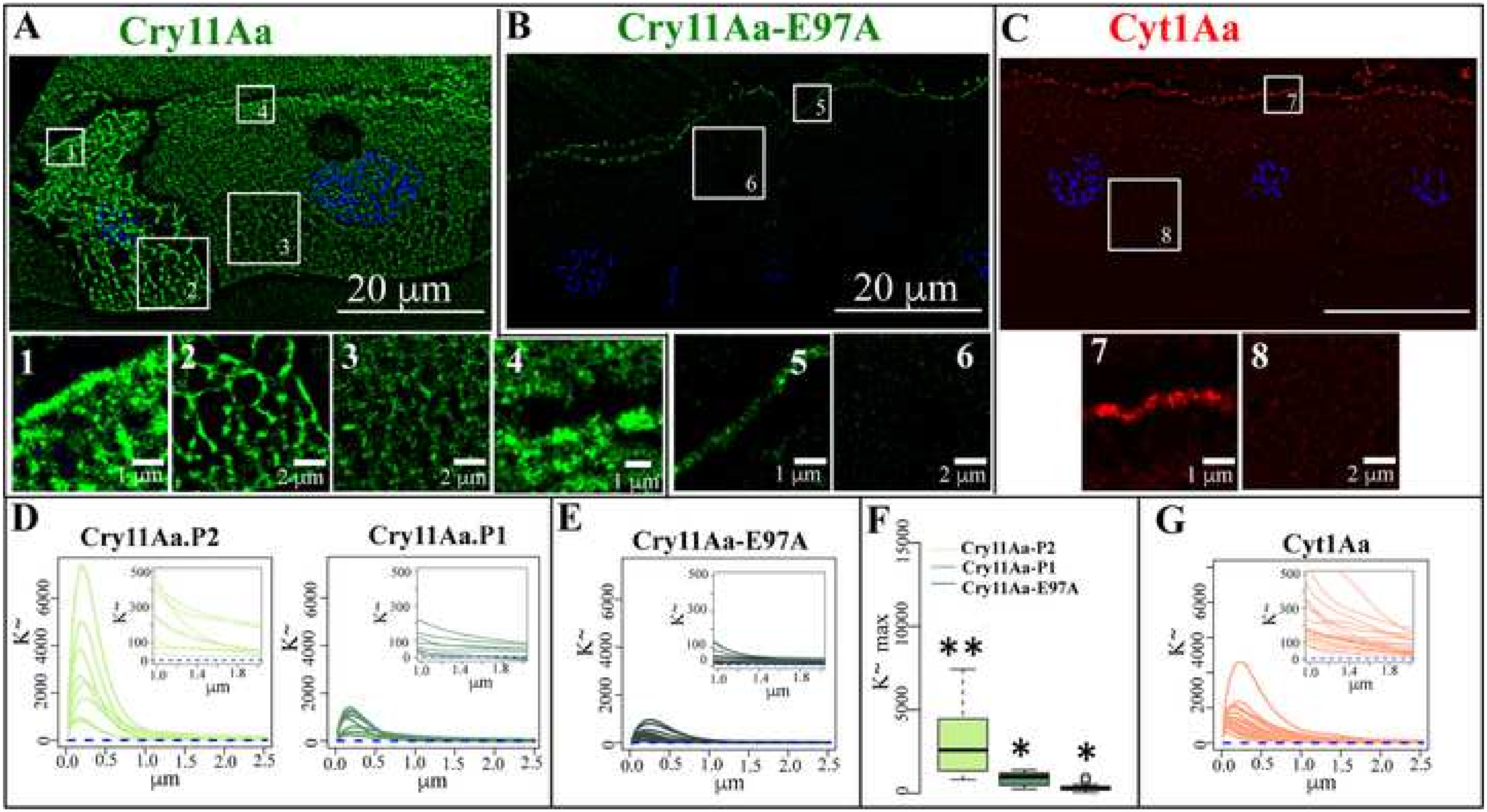

Figure 7

Click here to access/download;Figure;Figure 7.tif

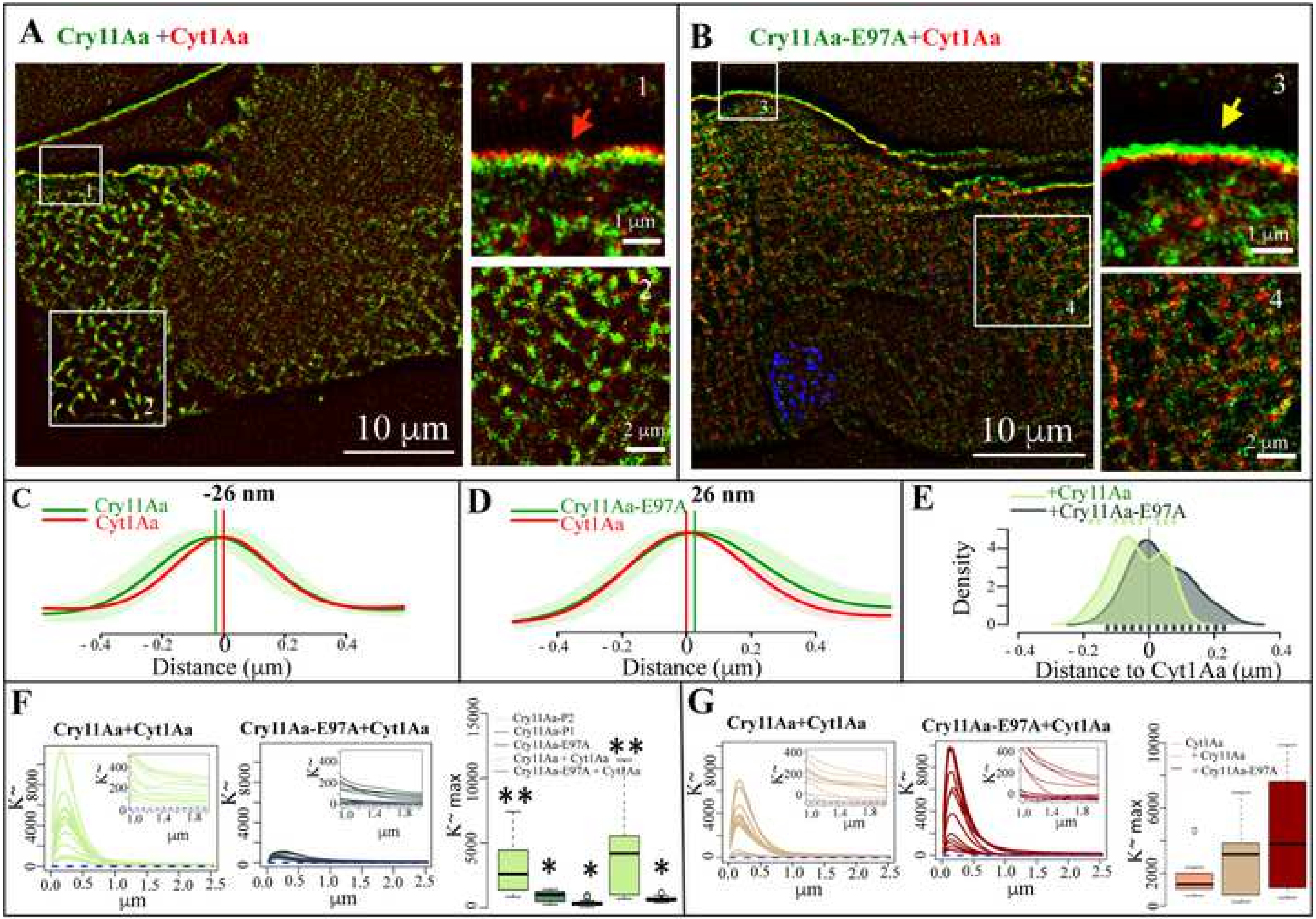

Click here to access/download
Supporting Information
Supp material.docx
