# Supplementary material for: In vivo nanoscale analysis of the dynamic synergistic interaction of Bacillus thuringiensis Cry11Aa and Cyt1Aa toxins in Aedes aegypti
Source: PLoS Pathog. 2021 Jan 19;17(1):e1009199. doi: 10.1371/journal.ppat.1009199 (PMC7846010; doi:10.1371/journal.ppat.1009199)
Supplement: Attachment — Submitted filename: comentarios Revisores Final.docx [file ppat.1009199.s013.docx]

Responses to Reviewer's Questions

**Part I - Summary**

**Reviewer #1:** This comprehensive manuscript (highly significant) refines

a conventional paradigm explaining the mode of action of mosquitocidal

proteins Cry11Aa and Cyt1Aa. Several proposed remarks that would improve

the manuscript are included as sticky notes throughout the text in the

attached MS.

**Response:** We thank the reviewer for his/her positive comments and specific notes that helped us to improve our manuscript. We have taken in consideration all comments included in the sticky notes found in the PDF sent by the reviewer

**Reviewer #2:** This manuscript uses very high-resolution light microscopy

with labelled proteins to investigate the spatial orientation of Bt

proteins in the mosquito larva in vivo. This is a new application of

this powerful technique in the field of Bacillus thuringiensis. However,

presentation of results before the microscopy is confusing and

contradictory, unnecessarily so. Misleading statements are made that

have nothing to do with the insights from microscopy. If these are

cleaned up, this would be a valuable contribution to the field.

**Response:** We have carefully reviewed the text of the complete document improving clarity of all results and conclusions, and we deleted the misleading elements that were pointed by the reviewer

-------------------------

**Part II - Major Issues: Key Experiments Required for Acceptance**

**Reviewer #1:** None required.

**Reviewer #2:** Abstract, lines 30-33 are misleading. 1) soluble proteins

are in fact toxic. 2) no evidence that larvae stop feeding for any

reason is given. 3) whether larvae ingest nontoxic proteins has no

relation to the first two points. See discussion of 1) below.

**Response:** We edited these sentences of the abstract. Specifically, we deleted the statement that mentioned that soluble toxins were not toxic, since the reviewer is right saying that soluble toxin is toxic. Now it is stated as: “ when larvae were fed with solubilized proteins the larvae were not killed and toxins were not accumulated inside the larval gut. In contrast, when larvae were fed with soluble non-toxic mutant proteins these proteins were found inside the gut bound to microvilli”.

In relation with the stop feeding effect we found a previous publication reporting that different mosquito species indeed showed a stop feeding behavior after being fed with spore/crystals mixtures of Bti (Aly 1988). Specifically, after 1 h ingestion of Bti, *Aedes aegypti* larvae stops feeding. These studies were done by analyzing the ingestion of latex microspheres (Aly 1988). We discussed these data and added this reference in the introduction section of the corrected version.

Aly Ch. 1988 Filter feeding of mosquito larvae (Dipt., Culicidae) in the presence of the bacterial pathogen *Bacillus thuringiensis* var. *israelensis* J Appl Entomol 105:160-166

"Only crystal inclusions were toxic, which remain insoluble in the

gut-lumen for a prolonged time." The authors are implying that

solubility is detoxifying the crystal, which is incorrect.

**Response:** We deleted this sentence.

Information on the size of the crystals is necessary. What size of

particle is trapped by the mosquito larva's filter-feeding apparatus,

and what size of particle is not?

**Response**: The size of particles that are retained by mosquito larvae was estimated to be from 0.71 to 26 microm (Dadd 1971). The crystal inclusion of Bti or Cyt1Aa have a size of around 1 microm (Wu and Federici 1993; Tetreau et al 2020). This information was added in introduction section of the corrected version.

Dadd H 1971 Effects of Size and Concentration of Particles on Rates of Ingestion of Latex Particulates by Mosquito Larvae. Ann Entomol Soc Am 64:687-693

Wu D., Federici B 1993. A 20-Kilodalton Protein Preserves Cell Viability and Promotes CytA Crystal Formation during Sporulation in Bacillus thuringiensis J Bacteriol 175:5276-5280

Tetreau et al 2020. Serial femtosecond crystallography on in vivo-grown crystals drives elucidation of mosquitocidal Cyt1Aa bioactivation cascade. Nature communications 11: 1153 doi.org/10.1038/s41467-020-14894-w

line 131. The authors should add the information from Ref 21 that

soluble proteins attached to latex beads were trapped by the mosquito

larvae and killed them. Soluble proteins are toxic.

**Response:** We added information of Ref 21 in introduction as suggested.

The observation is not "reinforced" by the fact that mosquito larvae are

filter feeders. It is explained by that fact.

**Response:** We edited introduction. The word “reinforced” has been eliminated and now we explain there that mosquitoes are filter feeders and included the size of particles that are filter by the larvae. We also included the description of the previous work showing that solubilized toxin proteins adsorbed in latex bead retain toxicity.

The authors state that soluble material is excluded from the gut. This

is incorrect--it is not excluded from the gut. The concentration of

pre-solubilized proteins in the gut can be no greater than its

concentration in the medium. Thus the proteins are toxic but not at that

concentration. The authors would need to measure the concentration of

protein recently dissolved from trapped crystals in the midgut to know

what the toxicity of soluble proteins is.

**Response**: We have edited this paragraph. The reviewer is right in saying that “The concentration of pre-solubilized proteins in the gut can be no greater than its

concentration in the medium” . Here we are using a high toxin concentration of labeled solubilized protein (1000 ng per ml), so if the larvae ingest the toxin we should be able to observe it inside the gut, as was observed with the non-toxic mutants.

First, our data showed that this concentration of 1000 ng per ml of soluble protein did not kill the larvae after 24 h, in contrast with 1000 ng per ml of the crystals that were highly active and killed the larvae after 24 h of crystal ingestion.

Second, we found that inside the gut of the larvae we did not observe the labeled soluble toxins or found marginal labeled protein concentration. Figure S3 shows that only after 3 h after ingestion of labeled soluble toxin protein, some toxin protein was found bound to the microvilli of the posterior region. The other incubation times analyzed showed extremely low concentration of labeled soluble toxin or no soluble toxin inside the gut. The comparison of images of labeled soluble toxin in the midgut with those of crystal ingestion, show a clear difference in their detection and we hypothesized that these data might reflect larvae’s capacity to sense midgut damage’

Third, in the case of the mutant proteins that were inactive, we observed that at the same concentration (1000 ng per ml), these labeled soluble protein are found inside in the gut at all times analyzed (Fig 3 and S3). These data suggest that soluble labeled mutant toxins went inside the larval gut and were able to bind to receptors located in the microvilli of the cells. It is important to mention that Cry11Aa or Cyt1Aa mutants used here are single point mutations, that are not toxic because these mutated proteins are affected in pore formation activity but they were still able to bind to receptors or to their target membranes [see 26, 27].

For these reasons, we hypothesized that the larvae reduce ingestion the soluble toxins that are active and were causing damage to their gut tissue, explaining why their ingestion is weak, or not optimal compared to crystals and for this reason we were unable to detect labeled activated soluble proteins inside the gut. It is true that we did not measure ingestion and further studies about that should be done in the future. Thus, we edited the text to toned down all arguments stating that larvae stop feeding. As mentioned above, previously it was shown that *Aedes aegypti* larvae indeed stops feeding after ingestion of Bti spore/crystals mixture (Aly 1988). Thus, it is possible that ingestion of soluble active toxins triggers also a stop feeding response. However, this remains to be studied in the future.

The statements that crystals are toxic and soluble proteins are not

toxic are not correct. A crystal that does not form soluble pore-forming

proteins cannot kill the insect. Crystals per se are not toxic. Soluble

proteins form pores and kill the insect. Soluble proteins are toxic. The

authors are skipping over the filter-feeding aspect of mosquito larva

that causes particle-size-dependent concentration and this has led them

to make statements that are descriptions of the phenomena of what

happens when crystals or previously solubilized proteins are put in the

water. However these are not accurate statements about toxicity. The

data in the manuscript are valid, but the interpretation is

unnecessarily confusing.

**Response:** We totally agree with the reviewer and we have changed interpretation of our data all over the text. We agree that soluble toxins are active, that crystals are not toxic per se, and that they are toxic only after protoxins were solubilized and activated inside the larval gut. We propose here the hypothesis that the early damage in the midgut tissue potentially caused by the soluble toxin triggers feeding interruption. As the damage of crystal in gut tissue might be slower since it has to be solubilized in the gut. Larvae could ingest high quantities of crystals before sensing midgut damage and stopping feeding. We clearly understand that we did not measured rate of feeding in our assays and that remains to be analyzed in the future.

More explanation of Figure 2 is required. Exactly what region

corresponds to the caeca? The anterior midgut? the posterior midgut? are

there other regions not named? Is there a food bolus or was it removed?

Is there a peritrophic matrix or was it removed? There needs to be a

diagram showing the regions mentioned in the text. Since some images

were constructed from composites, the originals must be presented in the

Supplementary Material. What is the dark region in the middle? Is it a

food bolus surrounded by a peritrophic matrix? Is Figure 2B just showing

movement of the food bolus? In Figure 2D, comment on the appearance of

the midgut after 9 hours as well as the caeca. Does degradation of the

caeca cause a connection between the gut lumen and the body cavity, or

does the caeca just shrink in size?

**Response:** The *caeca* is composed of eight blind ending tubes, which empty into the anterior end of the midgut (Thompson, 1905), thus it is located before the anterior midgut region. We now added a supplemental figure (new Figure S1) showing a diagram of the larval gut structure as suggested by the reviewer. Moreover, we added labels of the control midgut in figure 2A, localizing the *caeca* and the three regions of the midgut.

- The food bolus was not removed when we dissected the midgut tissue, since it helps to have a complete structure of the tissue. This information was added to materials and methods.

- As indicated by the reviewer all original figures of composite images are now presented in Supplementary material (New Figs S8-S11).

- Yes, the darker region in the middle is the food bolus surrounded by the peritrophic matrix

- Figure 2B shows the morphology of the midgut tissue after ingestion of Cry11Aa crystals. This figure shows that after 24 h ingestion of high concentration of Cry11Aa-crystals, the *caeca* is severely affected, almost complete destroyed and disappeared.

- Figure 2D shows a more severe damage since after 3 h intoxication with a mixture of Cry11Aa+Cyt1Aa crystals. The *caeca* was severely affected and after 9 h the gastric *caeca* is completely destroyed. We commented about these morphological changes in the text of result section as indicated by the reviewer.

- With these images we can conclude that *caeca* shrink in size after Cry11Aa crystal ingestion, more studies are needed to describe if the connection with gut lumen is also affected.

We edited the text an added all these changes in result section of the manuscript.

Thompson, M T. 1905. Alimentary canal of the mosquito. Proc. Boston Soc. Nat. Hist., 32,145-202.

lines 183-184. Labelling proteins does not cause a mutation. Do the

authors want to compare mutant proteins with wild-type proteins, or

labelled proteins with unlabelled proteins?

**Response:** The reviewer is correct the labeling did not cause a mutation in the proteins. Here we wanted to compare activity of the labeled proteins with unlabeled proteins. This was just a control. We deleted this misleading sentence.

lines 189-195. The data do not support the authors' assertion that

larvae did not ingest soluble toxin. The data at 3 hours show that they

did ingest it, but that they later got rid of it or somehow changed the

membrane to make it less adhesive to the toxin.

**Response:** We edited this paragraph, stating that the toxin is ingested at early times of intoxication and that later the concentration of the toxin found inside the gut is marginal.

The authors need to set their results in the context of quantitative

measurements on how much the filter-feeding apparatus increases the

concentration of particles actually ingested, relative to the

concentration of particles in the water. There must be data available on

that somewhere, for some mosquito species. This should be a function of

particle size, or at least minimum particle size, and time (because more

particles will accumulate in the apparatus for longer times).

**Response:** As stated above we found information of the estimated particle size that is retained in the larval gut (from 0.71 to 26 microm) (Dadd 1971). This was included in the text of introduction.

We also found information supporting those larvae of *Ae. aegypti* abruptly ceased feeding 60–90 min post‐treatment with Bti crystals (Aly 1988). This was included in the text of discussion.

lines 217-221. This paragraph does not make sense. The authors claim

that wild-type Cry11Aa which could be toxic, is not toxic because it is

not ingested, and the mutant Cry11Aa-E97 is ingested because it could

not be toxic. This does not support the statement "the lack of toxicity

of Cry11Aa soluble proteins correlated with a strong reduction of

ingestion." This explanation does not make sense. Is the mosquito doing

something to get rid of the wild-type protein that it is not doing to

the mutant protein? And how repeatable is the observation in Figure 3A?

**Response:** This paragraph was deleted from results. Our hypothesis is that when larva ingested the soluble active toxin, the toxin start damaging the gut tissue, and larvae responded by reducing feeding filtration, as previously demonstrated for ingestion of active crystal inclusions (Aly 1988). This could explain the low detection of soluble toxin inside the gut of the larvae after 3, 6, 9 or 24 h (Fig 3 and S3). In contrast, when larvae were feed with soluble non-toxic Cry11Aa-E97A mutant, this protein was ingested but no response in reducing feeding filtration was produced since this mutant protein did not damage the larval gut. This hypothesis was included in discussion. It is important to recall that this is a point mutation where a single amino acid was changed. The overall characteristics of the mutant protein are similar to the wild type in relation to size, solubility, stability against protease treatment, binding to receptors etc., only oligomerization and pore formation activity was affected in this mutant protein, resulting in a non-toxic mutant protein. Also, similar results were obtained with Cyt1Aa and Cyt1AaV122E mutant, despite the fact that Cry11Aa and Cyt1Aa differ in size and mode of action.

A total of five larvae were dissected for each condition and each assay was performed at least three times. This information was added to the figure legend and materials and methods section. The supplemental figure S3 show representative images of midgut tissues at different times (3, 6, 9, 24 h) after intoxication with labeled Cry11Aa or Cry11Aa-E97 mutant, showing similar results as those presented after 24 h in figure 3A and 3B.

what is the principle of the labelling with the Alexa dyes? "According

to manufacturer's instructions" is not specific enough. Many different

functional groups could be targetted. When a crystal is labelled, how

much of the protein is labelled? Is only the protein on the outside of

the crystal labelled?

**Response**: We used AlexaFluor 546- succinimidyl ester (NHS) and AlexaFluor 647-NHS, that label the primary amines (R-NH 2) of the proteins, thus they specifically label Lys residues. This information was included in materials and methods.

Our data indicate that only the protein from the outside of the crystal inclusions was labelled, since labelling of crystal was 10 fold less efficient than labeling of the soluble proteins. These data are in accordance with the crystallography analysis of the crystals produced in Bti bacteria, that showed that crystals are highly compact (Tetreau et al 2020).

line 374, precludes us to derive conclusions > prevents us from making

conclusions. The words "precludes", "precluding" are used incorrectly

throughout the manuscript.

**Response:** These words were corrected

lines 246-247 the arbitrary designation P1 and P2 for cells without or

with label internalization is useless. Don't add to the abbreviations

the reader must keep in mind to understand the paper.

**Response:** We have edited the complete document and deleted designations of P1 and P2 cells.

lines 267-274. I do not understand the point of this paragraph and

Figure S4. Why is it surprising that labelled crystals can be seen in

the lumen? Why is it interesting that they get washed out or dissolved

over time? The authors state "the crystal inclusions may spend a long

period of time inside the larval gut", but most of them do not.

**Response:** We deleted figure S4 to avoid confusion. It was previously described that crystals are solubilized immediately after ingestion but we did not see that, since crystals remain in the gut lumen for quite long period of time.

line 278, 289-290 independent evidence, such as behavioral observations,

is required to conclude that the mosquito larvae stopped feeding. It

cannot be concluded with the data presented here.

**Response:** We deleted these sentences and modify the corresponding text regarding feeding behavior. We agree with the reviewer that more studies are required to be done in the future.

-------------------------

**Part III - Minor Issues**: Editorial and Data Presentation Modifications

**Reviewer #1:** See the sticky notes in the attached MS.

**Comments found in the sticky notes made by Reviewer 1:**

**Note 1,** Page 8: Replace by "Alkaline Phosphatase"(First time mentioned)

**Response:** We deleted this sentence in abstract but included alkaline phosphatase the first time that it was mentioned in the text as indicated

**Note 2,** page 8: Not "potential": feeding arrest is already a mode of toxicity.

- Do sub-lethal concentrations eventually cause arrest of develoment hence death or not?

**Response:** We edited this sentence since we really did not determine feeding arrest behavior. Yes sub-lethal concentrations caused slow down of larval development.

**Note 3,** page 9: Please, stick to the new numenclature posted on https://www.bpprc.org , and described in <https://orca-mwe.cf.ac.uk/133399/1/1-s2.0-S0022201120301440-main.pdf>

**Response**: We edited the text mentioning the new nomenclature and we added the corresponding reference.

**Note 4,** page 12: Improve this sentence

**Response**: This sentence was edited as indicated.

**Note 5,** pages 15, 17, 20, 21, 22, 23, 51, 52, 53 and 54: Should be "inset" (with no 'r')!

**Response:** All these notes were corrected in the text.

**Note 6,** page 21. Shouldn't it be Fig 4C!?

**Response:** Figure 4 shows the distribution of Cry11Aa. Binding to microvilli from the different midgut regions is much better described in figure 4A and cell internalization was more clearly analyzed in figure 4C. Thus, we added here Fig 4A and 4C.

**Note 7,** page 22: And what about insets 7 and 8 as well?

**Response**: These insets are now more clearly described in the text.

**Note 8,** page 27: Why not mention the detergent-like mechanism of Cyt1A?

**Response:** We added a sentence talking about the detergent-like mechanism of Cyt1A

**Note 9**, page 28: This observation may imply detergent like mechanism of Cyt1Aa

**Response:** We edited this sentence and indicated that this observation may support the detergent like mechanism of Cyt1Aa

**Note 10**, page 31: Add "expressing the"

**Response**: Corrected

**Note 11**, page 31: add "separately"

**Response**: Corrected

**Reviewer #2**: line 70. "particularity" is not a word in English. In

general, there are several errors in English which I would have expected

to be corrected by the coauthor with the greatest familiarity with the

language.

**Response:** This word was deleted from the manuscript

For each figure showing something labelled with a color, the description

of all of the colors and what they label must be in the caption for that

figure. Red, green, and blue.

**Response:** Description of red, green, and blue colors were included in all figure legends

The SRRF Microscopy is not a standard technique, and the authors have

tried to explain the methods. But the point of using this technique is

lost in the details. The authors should explain the method in terms

understandable to a scientist not familiar with the technique, in such a

way as to explain why it is being used.

**Response:** We edited the text and we are presenting now a clearer explanation of how it works in results and discussion sections. However, since this is not a standard technique we think it is important to present a clear and detailed explanation in materials and methods section. We also edited materials and methods section to improve clarity.

line 687 why temporal analysis? What is changing over time?

**Response:** In the present study samples where fixed with 4% paraformaldehyde supplemented with 5% sucrose in PBS pH 7.4. For these tissues we took 100 confocal sequence of images collected at the same imaging plane. Hence, this temporal image stack of 100 confocal images harbors dynamics of the photophysical transitions experienced by the fluorophores due to the fluorescence excitation regime, at single pixel level. The temporal analysis of such transitions is required to be analyzed to overcome the diffraction limit of light, hence, to increase the resolution of the final reconstructed image. This explanation has been incorporated in material and methods.

line 754 state in words what the K-function is supposed to measure, and

what it tells us about the distribution of particles. The purpose of the

technique is getting lost in the description of the method.

**Response**: The Ripley’s K~ function computes the degree of organization of a distribution of points by assessing the density of information at all radial distances. Our data revealed a remarkable degree of organization of labelled Cry11Aa within the cytoplasm of Cry11Aa-brilliant cells. The entire section of spatial statistics was edited to keep focus on the meaning of the performed study, for the sake of clarity; much of the mathematical load has been removed form materials and method section.

line 755-757 a diagram would be helpful here.

**Response:** Figures 6D, 6F, 6G 7F and 7G correspond to the requested K plots.

line 768 please give an intuitive explanation of why edge effects

disrupt the K-function calculation, and how Besag's correction solves

this problem.

**Response:** The K-Ripley´s function computes the degree of organization of a distribution of fluorescent points by assessing the density of fluorescent information at all radial distances. Images presented edge issues when commuting the K statistics due to the lack of information outside of the spatial domain of the image. This means that it is not possible to assess the organization of the data near the boundaries of the image due to the lack of information outside of the image. Besag’s correction implements set theory to compensate such bias by proposing a normalization equation that considers proximity to the edges of the image. This was included in the text.

lines 783-789 this discription is helpful. But what does it mean for the

image analysis? Are we only interested in clustered distributions? If

so, why?

**Response:** We have improved such description by linking its meaning to the corresponding indications of randomness shown at the $\tilde{K}$panels of Figs 6 and 7. We are not only interested in clustered distributions, but rather to assess the degree of organization of Bt toxin within the cells of the larval gut tissue.

line 314, the word "awry" is never used as an adjective in English.

**Response:** This word was deleted

The abbreviation IF is defined once. This is a useless abbreviation. Use

the words "fluorescence intensity" the first time, and the word

"fluorescence" subsequently.

**Response**: IF abbreviation was deleted from the manuscript

Line 346. What are "tents of cells"?

**Response:** We edited the text, it means from 10 to 20 individual cells

line 403. is 26 nm the distance between the two proteins, or the

distance between the two labels on the two proteins? Does this tell us

anything about the orientation of the proteins?

**Response:** It indicates the average distance between the two proteins distributions. This study assesses the organization of distributions of both Bt toxins, rather than the organization of single molecules, we are no able to develop interpretations about the orientation of single proteins. Further studies will need to be done to describe their relative orientation.

line 441 it is not clear what comparisons are described here. what

pattern is not significantly different from what other pattern?

**Response:** Here we are talking about the distribution of Cyt1Aa when it was used in a mixture with Cry11Aa or in a mixture with Cry11AaE97A shown in Figure 7G. We edited the text.

line 451-452. The statement of reduced ingestion can only be supported

by behavioral observations, not the evidence given in the manuscript.

**Response:** We deleted this sentence**.**

line 466 If the authors are tempted to propose such an unreasonable

hypothesis, they must state how it could be tested. Or, more likely,

that it cannot be tested, given that many other Bt toxins are found in

crystals of protoxins, which are consumed by lepidoptera or coleoptera.

**Response:** We deleted these sentences.

line 525. The authors cite Ref. 39 which shows that RAB-5 mediated

endocytosis defends nematodes against Cry5Ba damage. Could the

RAB-11-dependent shedding of microvilli shown in the same paper be

responsible for the destruction of the caecum?

**Response:** This is an interesting possibility, however, it is difficult to say since shedding of microvilli does not involve cell destruction. The nematodes do not have a *caeca* structure. More studies need to be done in the future in order to describe the specific process that is responsible of cell break down observed in the mosquito midgut tissue after Cry11Aa ingestion.

line 539 the explanation of the "net" structure within the cytoplasm is

not convincing.

**Response:** We deleted this explanation in this paragraph.

The orientation of Cyt1Aa and Cry11Aa layers on the microvilli is very

interesting. The authors should explicitly state that it could only be

observed using the SRRF technique.

**Response:** This is now clearly stated in the text as suggested by the reviewer

-------------------------

Figure Files:

While revising your submission, please upload your figure files to the

Preflight Analysis and Conversion Engine (PACE) digital diagnostic tool,

https://pacev2.apexcovantage.com [3]. PACE helps ensure that figures

meet PLOS requirements. To use PACE, you must first register as a user.

Then, login and navigate to the UPLOAD tab, where you will find detailed

instructions on how to use the tool. If you encounter any issues or have

any questions when using PACE, please email us at figures@plos.org.

Data Requirements:

Please note that, as a condition of publication, PLOS' data policy

requires that you make available all data used to draw the conclusions

outlined in your manuscript. Data must be deposited in an appropriate

repository, included within the body of the manuscript, or uploaded as

supporting information. This includes all numerical values that were

used to generate graphs, histograms etc.. For an example see here on

PLOS Biology:

http://www.plosbiology.org/article/info%3Adoi%2F10.1371%2Fjournal.pbio.1001908#s5.

Reproducibility:

To enhance the reproducibility of your results, PLOS recommends that you

deposit laboratory protocols in protocols.io, where a protocol can be

assigned its own identifier (DOI) such that it can be cited

independently in the future. For instructions see

http://journals.plos.org/plospathogens/s/submission-guidelines#loc-materials-and-methods

[4]

-------------------------

In compliance with data protection regulations, you may request that we

remove your personal registration details at any time. (Remove my

information/details) [5]. Please contact the publication office if you

have any questions.

Links:

------

[1] https://journals.plos.org/plospathogens/s/editorial-and-peer-review-process#loc-peer-review-history

[2] https://www.plos.org/privacy-policy

[3] https://pacev2.apexcovantage.com/

[4] http://journals.plos.org/plospathogens/s/submission-guidelines

[5] https://www.editorialmanager.com/ppathogens/login.asp?a=r
